# Identification of diverse defense mechanisms in rainbow trout red blood cells in response to halted replication of VHS virus

**DOI:** 10.12688/f1000research.12985.2

**Published:** 2018-02-09

**Authors:** Ivan Nombela, Sara Puente-Marin, Veronica Chico, Alberto J. Villena, Begoña Carracedo, Sergio Ciordia, Maria Carmen Mena, Luis Mercado, Luis Perez, Julio Coll, Amparo Estepa, Maria del Mar Ortega-Villaizan

**Affiliations:** 1 Instituto de Biología Molecular y Celular, Universidad Miguel Hernández, Elche, Spain; 2 Área de Biología Celular, Departamento de Biología Molecular, Universidad de León, León, Spain; 3 Unidad de Proteómica, Centro Nacional de Biotecnología, Madrid, Spain; 4 Instituto de Biología, Pontificia Universidad Católica de Valparaíso, Valparaíso, Chile; 5 INIA-SIGT–Biotecnología, Madrid, Spain

**Keywords:** nucleated red blood cells, rainbow trout, VHSV, rhabdovirus, immune response, antiviral

## Abstract

**Background:** It has been described that fish nucleated red blood cells (RBCs) generate a wide variety of immune-related gene transcripts when viruses highly replicate inside them and are their main target cell. The immune response and mechanisms of fish RBCs against viruses targeting other cells or tissues has not yet been explored and is the objective of our study.

**Methods:** Rainbow trout RBCs were obtained from peripheral blood, ficoll purified and exposed to *Viral Haemorrhagic Septicaemia virus* (VHSV). Immune response was evaluated by means of RT-qPCR, flow cytometry, immunofluorescence and isobaric tag for relative and absolute quantification (iTRAQ) protein profiling.

**Results:** VHSV N gene transcripts incremented early postexposure and were drastically decreased after 6 hours postexposure (hpe). The expression of type I interferon ( *ifn1*) gene was significantly downregulated at early postexposure (3 hpe), together with a gradual downregulation of interferon-inducible *mx *and *pkr *genes until 72 hpe. Type I IFN protein was downregulated and interferon-inducible Mx protein was maintained at basal levels. Co-culture assays of RBCs, previously exposed to UV-inactivated VHSV, and TSS (stromal cell line from spleen) revealed IFN crosstalk between both cell types. On the other hand, anti-microbial peptide β-defensin 1 and neutrophil chemotactic factor interleukin 8 were slightly upregulated in VHSV-exposed RBCs. iTRAQ profiling revealed that VHSV exposure can induce a global protein downregulation in rainbow trout RBCs, mainly related to RNA stability and proteasome pathways. Antioxidant/antiviral response is also suggested to be involved in the response of rainbow trout RBCs to VHSV.

**Conclusions:** A variety of mechanisms are proposed to be implicated in the antiviral response of rainbow trout RBCs against VHSV halted infection. Ongoing research is focused on understanding the mechanisms in detail.

## Introduction

Fish are the most primitive vertebrates possessing many of the immune system cells (lymphocytes, NK cells, macrophages, etc) and molecules (interleukins, chemokins, receptors, etc) found in higher vertebrates. In contrast to higher vertebrates, however, fish lack bone marrow, lymph nodes, IgG-switch, and have tetrameric rather than pentameric IgM, with a more limited binding repertoire than mammals ^1^. Fish red blood cells (RBCs), the most abundant cell type in the blood, have receptors capable of recognizing pathogen associated molecular patterns and respond to them with differentially expressed cytokine transcripts ^2, 3^ and cytokine-like factors ^4^. Fish RBCs generate a wide variety of immune-related gene transcripts when viruses highly replicate inside them ^5– 7^, while their mammalian counterparts are unable to do this. In light of this evidence, an outstanding question is whether fish RBCs are able to respond to viral infections that are well known to replicate in other cells or tissues, and if they could further contribute with compensatory immune responses in order to physiologically combat viral infections that do not target RBCs.

To explore *in vitro* the above mentioned question, we used rainbow trout ( *Oncorhynchus mykiss*), an important aquacultured species, together with *Viral Haemorrhagic Septicemia virus* (VHSV), a rhabdovirus also called the ‘fish ebola’, which causes important losses of high economic impact on world-wide salmonid aquaculture ^8^. VHSV viruses are bullet-shaped enveloped virions with single-stranded negative-sense RNA with a genome of 11.2 kbp ^8– 10^. In rainbow trout, kidney and spleen endothelial cells are the first targets of VHSV. Afterwards, hematopoietic elements of kidney and spleen undergo necrosis and degeneration, most specifically at melanomacrophage centers (reviewed in Kim and Faisal ^11^). However, there are no references for VHSV targeting specifically RBCs, therefore represent a good model to investigate the immune response of RBCs to viruses targeting other cells or tissues. VHSV cell entry has been described to be mediated by binding initially to fibronectin, an abundant glycoprotein of the extracellular matrix, allowing then VHSV to bind to the cells via integrin receptors and enter by fusion or endocytosis ^12^.

In this study, we describe how *in vitro* cultures of rainbow trout RBCs upregulated the expression of some immune proteins as part of their antiviral immune response against VHSV, whose infection appeared to be halted in rainbow trout RBCs. Simultaneously, interferon-inducible *mx* and *pkr* genes showed a downregulation tendency during VHSV early replication, after 6 hpe. In addition, protein levels corresponding to BD1 (β-defensin 1 – an anti-microbial peptide known to be involved in antiviral innate immunity ^13, 14^– and IL8 (Interleukin 8 – a neutrophil chemotactic factor–), are shown, to our knowledge, for the first time, as characteristic of rainbow trout RBCs antiviral immune protein responses. Further, iTRAQ-based protein profiling of VHSV-exposed RBCs showed a global protein downregulation, mainly related to RNA stability and proteasome pathways. Related to this fact, phosphorylation of the α-subunit of translational initiation factor 2 (eIF2α) and protein synthesis inhibition could be implicated in the inhibition of VHSV replication and RBCs proteome shut-off. Also, antioxidant related antiviral response is also suggested as involved in the response of rainbow trout RBCs to VHSV halted infection. In summary, we suggest a wide range of mechanisms implicated in the antiviral response of rainbow trout RBCs against VHSV halted infection.

## Methods

### Animals

Rainbow trout ( *Oncorhynchus mykiss*) individuals of approximately 5–6 cm were obtained from a VHSV-free commercial farm (PISZOLLA S.L., CIMBALLA FISH FARM, Zaragoza, Spain), and maintained at University Miguel Hernandez (UMH) facilities at 14°C, with a re-circulating dechlorinated-water system, at a stocking density of 1fish/3L, and fed daily with a commercial diet (SKRETTING, Burgos, Spain). Prior to experiments, fish were acclimatized to laboratory conditions over 2 weeks. The number of individuals used is indicated by an “n” in each experiment.

### Antibodies

Rabbit polyclonal antibodies against rainbow trout β-defensin (BD1) (RRID: AB_2716268) (unpublished, Figure S1) and rainbow trout Mx3 (RRID: AB_2716267) ^15– 17^ were produced at the laboratory of Dr. Amparo Estepa. Mouse polyclonal antibodies against rainbow trout IL1β (RRID: AB_2716269) ^18, 19^, IL8 (RRID: AB_2716272) ^20^, TNFα (RRID: AB_2716270) ^21^, Hepcidin (RRID: AB_2716273) ^22^, NKEF (RRID: AB_2716271) ^23, 24^, IFNγ (RRID: AB_2716275) (unpublished, Figure S2A) and IFN1 (RRID: AB_2716274) (unpublished, Figure S2B) were produced at the laboratory of Dr. Luis Mercado. Rabbit polyclonal antibody against human NF-κβ p65 antibody (Cat#ab7970, RRID: AB_306184) was purchased from AbCam (Cambridge, UK). This p65 antibody epitope corresponds to the C-terminal region of the p65 protein, similarly to other p65 antibodies used for teleost species ^25– 27^. To label VHSV, we used the mouse monoclonal 2C9 antibody (RRID: AB_2716276) ^28^ against the N protein of VHSV (N _VHSV_) produced at Dr. Coll’s laboratory. Anti-Rabbit IgG (H+L) CF™ 488 antibody produced in goat and Anti-Mouse IgG (H+L) CF™ 488 antibody produced in goat were used as secondary antibodies (Sigma-Aldrich, Madrid, Spain). Rabbit polyclonal antibody against human eIF2α-P (Cat# E2152, RRID:AB_259283) and rabbit polyclonal antibody against human α-Actin (Cat#2066, RRID:AB_476693) were purchased from Sigma-Aldrich and used for western blotting.

### Cell cultures and virus

Rainbow trout RBCs were obtained from peripheral blood of fish sacrificed by overexposure to tricaine (tricaine methanesulfonate, Sigma-Aldrich; 0.2 g/l). Peripheral blood was sampled from the caudal vein using insulin syringes (NIPRO, Bridgewater, NJ). Blood samples were placed in a 2 ml eppendorf with RPMI-1640 medium (Dutch modification) (Gibco, Thermo Fischer Scientific Inc., Carlsbad, CA) supplemented with 10% FBS (fetal bovine serum) gamma irradiated (Cultek, Madrid, Spain), 1 mM pyruvate (Gibco), 2 mM L-glutamine (Gibco), 50 µg/mL gentamicin (Gibco) and 2 µg/mL fungizone (Gibco), 100 U ml−1 penicillin and 100 μg ml−1 streptomycin (Sigma-Aldrich). Then, RBCs were purified by two consecutive density gradient centrifugations (7206g, Ficoll 1.007; Sigma-Aldrich). Purified RBCs were cultured in the above indicated medium at a density of 5·10 ^5^ cells/ml in 24-well cell culture plates at 14°C.

The fish cell lines TSS, RTG-2 and EPC, were also used in this work. TSS (Trout Stroma from Spleen) ^29^ was donated by the laboratory of Dr. AJ Villena. TSS cells were maintained at 21°C in RPMI medium containing 20% FBS, 1 mM pyruvate, 2 mM L-glutamine, 50 µg/mL gentamicin and 2 µg/mL fungizone. RTG-2 (Rainbow Trout Gonad-2) cell line was purchased from the American Type Culture Collection (ATCC, 50643). RTG-2 cells were maintained at 21°C in MEM medium (Sigma-Aldrich) containing 10% FBS, 1 mM pyruvate, 2 mM L-glutamine, 50 µg/mL gentamicin and 2 µg/mL fungizone. EPC ( *Epithelioma Papulosum Cyprini*) ^30^ cell line was purchased from the ATCC (CRL-2872). Cells were maintained at 28°C, in RPMI-1640 10% FBS, 1 mM pyruvate, 2 mM L-glutamine, 50 µg/mL gentamicin and 2 µg/mL fungizone.

*Viral haemorrhagic septicaemia* virus (VHSV-07.71) ^31^, isolated in France from rainbow trout, was purchased from the American Type Culture Collection (ATCC, VR-1388) and propagated in EPC cells at 14°C, as previously reported ^32^. Supernatants from VHSV-infected EPC cell monolayers were clarified by centrifugation at 4000 x g during 30 min and kept at -80 °C. The virus stock was titrated in 96-well plates using an immunostaining focus assay ^33^. Clarified supernatants were used for the experiments at the indicated dilutions.

### Viral exposure assays

RBCs and RTG-2 cells were infected with VHSV at different multiplicities of infection (MOI), at 14°C. After 3 hours of incubation for RBCs and 1.5 hours for RTG-2, cells were washed with cold RPMI, then RPMI 2% FBS was added and infection incubated at 14°C, at the different times indicated for each assay. In the case of the time-course assay, the virus was not removed.

Virus titers present in VHSV-exposed RBCs supernatants were determined by plaque assays. Briefly, different dilutions of the supernatants (from 10 ^-1^ to 10 ^-4^) were added to EPC cell monolayers, grown in 24-well plates, at 14°C for 90 minutes. Then, culture media were removed and infected cell monolayers covered with a solution of RPMI-1640 cell culture medium with 2% FBS and a 2% aqueous solution of methyl cellulose (Sigma-Aldrich). Cell plates were incubated at 14°C for 5 days and then media with methyl cellulose were removed. Finally, EPC cell monolayers were stained with crystal violet-formalin to count plaques. Virus titers were expressed as plaque forming units (PFU) per ml.

Separately, N _VHSV_ RT-qPCR was also used to quantify viral RNA inside VHSV-exposed RBCs.

### Blocking of endosome acidification by NH _4_Cl

To block endosomal low-pH, NH _4_Cl (Sigma-Aldrich) at 7 mM was added to RBCs during VHSV exposure, which was carried out as described in the previous section. No significant cell death was observed in RBCs treated with NH _4_Cl, since the concentration used is known as non-cytotoxic in EPC ^33^ and RTG-2 ^17^ cells, but effective for reducing VHSV infectivity by 40% ^33^. After incubation period, viral titer in supernatants was calculated as described in the previous section.

### Neuraminidase treatment assay

Ficoll purified RBCs were pre-treated with 50 and 100 mU/ml of neuraminidase from *Vibrio cholerae* (Sigma-Aldrich), at 21°C for 30 minutes, before virus inoculation. After treatment, RBCs were washed once with PBS in order to completely remove the enzyme. After that, pre-treated cells were inoculated with VHSV at MOI 1. RBCs inoculated with UV-inactivated VHSV were used as control. UV-inactivated VHSV was generated by exposure to UV-B at 1 J/cm2 using a Bio-Link Crosslinker BLX E312 (Vilber Lourmat, BLX-E312), as previously described ^34^. Infection was monitored by RT-qPCR of N _VHSV_ gene 3 at 72 hpe.

### Co-culture assay

One day prior to co-culture, RBCs, extracted and seeded as indicated before, were stimulated using UV-inactivated VHSV over 24 hours. Subsequently, RBCs were washed once with cold RPMI and added to Corning® Transwell® polyester membrane cell culture inserts of 0.4 µm pore size (Corning, Sigma-Aldrich) on 24 well plates with previously cultured confluent TSS cells in RPMI 20% FBS. Co-culture was maintained for 24 hours at 14°C in RPMI 2% FBS. After that, cells were washed and stored in the indicated buffer and conditions for RNA extraction.

Separately, RTG-2 cells were treated with UV-inactivated VHSV, MOI 1, during 24 hours, at 14°C, in RPMI 2% FBS. After that, RTG-2 cell monolayers were washed once with cold PBS and cultured for 24h in RPMI 2%FBS fresh medium. This conditioned medium was used to stimulate rainbow trout RBCs, during 24h. After that, RBCs were washed and stored in the indicated buffer and conditions for RNA extraction.

### RNA isolation and cDNA synthesis

E.Z.N.A. ® Total RNA Kit (Omega Bio-Tek, Inc., Norcross, GA) was used for total RNA extraction in accordance with manufacturer’s instructions. Isolated RNAs were stored at −80°C until used. DNAse treatment was done in order to eliminate residual genomic DNA using TURBO™ DNase (Ambion, Thermo Fischer Scientific Inc.), following manufacturer’s instructions. RNA was quantified with a NanoDrop® Spectrophotometer (Nanodrop Technologies, Wilmington, DE). M-MLV reverse transcriptase (Invitrogen, Thermo Fischer Scientific Inc.) was used to obtain cDNA, as previously described ^35^. For viral messenger RNA (mRNA) quantitation, cDNA was obtained as described in 36.

### RT-qPCR and gene expression

Real-Time Quantitative PCR (RT-qPCR) was performed using the ABI PRISM 7300 System (Applied Biosystems, Thermo Fischer Scientific Inc.). Reactions were performed in a total volume of 20 μl comprising 12 ng of cDNA, 900 nM of each primer, 10 μl of TaqMan universal PCR master mix (Applied Biosystems, Thermo Fischer Scientific Inc.) with 300 nM of probe or 10 μl of SYBR green PCR master mix (Applied Biosystems, Thermo Fischer Scientific Inc.). Cycling conditions were 50°C for 2 min and 95°C for 10 min, followed by 40 cycles at 95°C for 15 s and 60°C for 1 min. Primers and probes used are listed in Table 1.

**Table 1.** Primer and probe sequences.

Gene	Forward primer (5’ – 3’)	Reverse primer (5’ – 3’)	Probe (5’ – 3’)	Reference or accession number
*ef1α*	ACCCTCCTCTTGGTCGTTTC	TGATGACACCAACAGCAACA	GCTGTGCGTGACATGAGGCA	97
*tlr3*	ACTCGGTGGTGCTGGTCTTC	GAGGAGGCAATTTGGACGAA	CAAGTTGTCCCGCTGTCTGCTCCTG	NM_001124578.1
*irf7*	CCCAGGGTTCAGCTCCACTA	GGTCTGGCAACCCGTCAGT	TCGAGCCAAACACCAGCCCCT	AJ829673
*ifn1*	ACCAGATGGGAGGAGATATCACA	GTCCTCAAACTCAGCATCATCTATGT	AATGCCCCAGTCCTTTTCCCAAATC	AM489418.1
*mx1-3*	TGAAGCCCAGGATGAAATGG	TGGCAGGTCGATGAGTGTGA	ACCTCATCAGCCTAGAGATTGGCTCCCC	98
*pkr*	GACACCGCGTACCGATGTG	GGACGAACTGCTGCCTGAAT	CACCACCTCTGAGAGCGACACCACTTC	NM_001145891.1
*il15*	TACTATCCACACCAGCGTCTGAAC	TTTCAGCAGCACCAGCAATG	TTCATAATATTGAGCTGCCTGAGTGCCACC	XM_021575070.1
*vig1*	CTACAATCAAGGTGGTGAACAATGT	GTGGAAACAAAAACCGCACTTATA	TCTCAAGCTTCGGCAACTCCAAGCA	XM_021582972.1
*hepcidin*	TCCCGGAGCATTTCAGGTT	GCCCTTGTTGTGACAGCAGTT	AGCCACCTCTCCCTGTGCCGTTG	AF281354.1
*β-globin*	CAACATCTTGGCCACATACAAGTC	TTGTCAGGGTCGACGAAGAGT		NM_001160555.2
*fth*	GGCGTATTACTTCGATCGTGATG	CCCTCCCCTCTGGTTCTGA		EU302524.1
*gstp1*	CCCCTCCCTGAAGAGTTTTGT	GCAGTTTCTTGTAGGCGTCAGA		BT048561.1
*nkef*	CGCTGGACTTCACCTTTGTGT	ACCTCACAACCGATCTTCCTAAAC		U27125.1
*sod1*	GCCGGACCCCACTTCAAC	CATTGTCAGCTCCTGCAGTCA		AF469663.1
*trx*	AGACTTCACAGCCTCCTGGT	ACGTCCACCTTGAGGAAAAC		XM_021614924.1
*N _VHSV_*	GACTCAACGGGACAGGAATGA	GGGCAATGCCCAAGTTGTT	TGGGTTGTTCACCCAGGCCGC	35

Gene expression was analyzed by the 2 ^-ΔCt^ or 2 ^−ΔΔCt^ method ^37^ where 18S rRNA or *ef1α* gene (Applied Biosystems, Thermo Fischer Scientific Inc.) were used as endogenous control.

### Intracellular stain and flow cytometry

RBCs were fixed with 4% paraformaldehyde (PFA; Sigma-Aldrich) in RPMI medium for 20 minutes. Permeabilization of RBCs was done in a 0.05% saponin (Sigma-Aldrich) buffer for 15 minutes. Primary antibodies were diluted in permeabilization buffer at recommended dilutions and incubated for 60 minutes at RT. Secondary antibodies were incubated for 30 minutes at RT. After every antibody incubation, RBCs were washed with permeabilization buffer. Finally, RBCs were kept in PFA 1% in PBS. For nuclear staining, RBCs were stained with 1 µg/mL of 4′-6-Diamidino-2-phenylindole (DAPI; Sigma-Aldrich) for 5 minutes. RBCs were analyzed by flow cytometry (FC) in a BD FACSCanto™ (BD Biosciences) flow cytometer. Immunofluorescence (IF) images were performed in an IN Cell Analyzer 6000 Cell Imaging system (GE Healthcare, Little Chalfont, UK).

### Protein digestion and tagging with iTRAQ 4plex ^TM^ reagent

Two pools of eight samples (two control: C1 and C2, and two VHSV-exposed (MOI 1, 14°C, 72 hpe): V1 and V2), with 8·10 ^6^ cells per sample, were used for iTRAQ 4plex protein profiling.

Pools, containing 6.4·10 ^7^ cells, were pelletized by centrifugation (5 min, 700 × g). Supernatant was carefully removed and RBC pellets (∼70–100 µL) were mixed with 250 µL of deionized water and frozen at – 80°C for 3 h. After thawing the lysate, it was centrifuged at 17000 × g for 20 min at 4°C to separate cytosolic supernatant and pelleted membrane fractions, as described in Puente-Marin *et al.* (unpublished report, Puente-Marin S, Nombela I, Ciordia S, Mena MC, Chico V, Coll J, and Ortega-Villaizan M). Subsequently, a new proteomic analysis method was carried out that combines fractionation into cytosolic and membrane fractions, haemoglobin removal of cytosolic fraction, protein digestion, pH reversed-phase peptide fractionation and finally LC ESI-MS/MS analysis of each fraction, as described in Puente-Marin *et al.* (unpublished report, as before). Briefly, haemoglobin of cytosolic fraction was removed using HemoVoid ^TM^ kit (Biotech Support Group, Monmouth Junction, NJ), following manufacturer instructions ^38^. For protein digestion of each fraction, 120 µg from haemoglobin-depleted cytosolic fraction were digested in chaotropic buffer, and 40 µg of membrane fraction was precipitated by methanol/chloroform method and re-suspended in 20 µl of chaotropic buffer. Digested samples (membrane and cytosol separately) were subsequently labelled using iTRAQ-4plex Isobaric Mass Tagging Kit (SCIEX), according to manufacturer's instructions as follows: 114, C1 (Pool control 1); 115, V1 (Pool VHSV-exposed 1); 116, C2 (Pool control 2); 117, V2 (Pool VHSV-exposed 2). Then, offline high pH reversed-phase peptide fractionation of the peptides from cytosolic fraction was performed on a SmartLine (Knauer, Berlin, Germany) HPLC system using an XBridge C18 column (100 × 2.1 mm, 5 μm particle; Waters, Milford, MA). Thirty fractions were collected and then pooled alternatively into 5 fractions. After labelling, samples were pooled, evaporated to dryness and stored at -20°C until LC−MS analysis.

### Liquid chromatography and mass spectrometry analysis (LC-MS)

A 1 µg aliquot of labelled mixture was subjected to 1D-nano LC ESI-MSMS (Liquid Chromatography Electrospray Ionization Tandem Mass Spectrometric) analysis using a nano liquid chromatography system (Eksigent Technologies nanoLC Ultra 1D plus, SCIEX,) coupled to high speed Triple TOF 5600 mass spectrometer (SCIEX) with a Nanospray III source. The analytical column used was a silica-based reversed phase Acquity UPLC® M-Class Peptide BEH C18 Column, 75 µm × 150 mm, 1.7 µm particle size and 130 Å pore size (Waters Corporation, Milford, MA). The trap column was a C18 Acclaim PepMap ^TM^ 100 (Thermo Fischer Scientific), 100 µm × 2 cm, 5 µm particle diameter, 100 Å pore size, switched on-line with the analytical column. The loading pump delivered a solution of 0.1% formic acid in water at 2 µl/min. The nano-pump provided a flow-rate of 250 nl/min and was operated under gradient elution conditions. Peptides were separated using a 250 minutes gradient ranging from 2% to 90% mobile phase B (mobile phase A: 2% acetonitrile, 0.1% formic acid; mobile phase B: 100% acetonitrile, 0.1% formic acid). Injection volume was 5 µl.

Data acquisition was performed with a TripleTOF 5600 System (SCIEX). Data was acquired using an ionspray voltage floating, 2300 V; curtain gas, 35; interface heater temperature, 150; ion source gas 1, 25; declustering potential, 150 V. All data was acquired using information-dependent acquisition (IDA) mode with Analyst TF 1.7 software (RRID: SCR_015785) (SCIEX). For IDA parameters, 0.25 s MS survey scan in the mass range of 350–1250 Da were followed by 30 MS/MS scans of 150ms in the mass range of 100–1800. Switching criteria were set to ions greater than mass to charge ratio (m/z) 350 and smaller than m/z 1250 with charge state of 2–5 and an abundance threshold of more than 90 counts (cps). Former target ions were excluded for 20 s. IDA rolling collision energy (CE) parameters script was used for automatically controlling the CE.

### Proteomics data analysis and sequence search

MS/MS spectra were exported to MGF format using Peak View v1.2.0.3 (RRID: SCR_015786)(SCIEX) and searched using Mascot Server v2.5.1 (RRID:SCR_014322)(Matrix Science, London, UK), OMSSA v2.1.9 ^39^, X!TANDEM 2013.02.01.1 ^40^, and Myrimatch v2.2.140 ^41^ against a composite target/decoy database built from the Oncorhynchus mykiss sequences at Uniprot/Swissprot Knowledgebase (available here, last update: 2017/01/26, 50.125 sequences), together with commonly occurring contaminants. Search engines were configured to match potential peptide candidates with mass error tolerance of 25 ppm and fragment ion tolerance of 0.02D, allowing for up to two missed tryptic cleavage sites and a maximum isotope error ( ^13^C) of 1, considering fixed methyl methanethiosulfonate modification of cysteine and variable oxidation of methionine, pyroglutamic acid from glutamine or glutamic acid at the peptide N-terminus, acetylation of the protein N-terminus, and modification of lysine, tyrosine and peptide N-terminus with iTRAQ 4-plex reagents. Score distribution models were used to compute peptide-spectrum match *P-*values ^42^, and spectra recovered by a FDR (False Discovery Rate) ≤ 0.01 (peptide-level) filter were selected for quantitative analysis. Approximately 1% of the signals with lowest quality were removed prior to further analysis. Differential regulation was measured using linear models ^43^, and statistical significance was measured using *q*-values (FDR). All analyses were conducted using Proteobotics software (Isobaric Tagging Analysis Workflow v.1.0, RRID:SCR_015787; Madrid, Spain). The cutoff for differentially regulated proteins was established at FDR *q*-value 5%.

### Pathway enrichment analysis

In order to evaluate the functionally grouped Gene Ontology (GO) and pathway annotation networks of the differentially expressed proteins, pathway enrichment analysis was performed using ClueGO (RRID:SCR_005748) ^44^ and CluePedia (RRID: SCR_015784) ^45^ Cytoscape plugins (Cytoscape v3.4.0, RRID:SCR_003032, ^46^). GO Biological process, GO Immunological process, KEGG (Kyoto Encyclopedia of Genes and Genomes), Wikipathways and Reactome functional pathway databases were used. A *P*-value ≤0.05 and Kappa score of 0.4 were considered as threshold values.

### Western blot assays

Control and VHSV-exposed RBCs cell pellets were resuspended in 30 µl of PBS with a cocktail of protease inhibitors (Sigma-Aldrich). Cells were then frozen/thawed 3 times and protein concentration adjusted before loading. Samples were loaded in Tris–Glycine sodium dodecyl sulfate 17% polyacrylamide gels under reducing conditions. Electrophoresis was performed at 100 V for 90 min. For blotting, proteins in the gel were transferred for 75 min at 100 V in transfer buffer (2.5 mM Tris, 9 mM glycine, 20% methanol) to nitrocellulose membranes (BioRad, Madrid, Spain). Membranes were then blocked with 8% dry milk, 1% Tween-20 in PBS and incubated with rabbit polyclonal antibody against human eIF2α-P (36.1 KDa) or rabbit polyclonal antibody against human α-Actin (42 KDa,) in PBS containing 0.5% dry milk, and 0.5% Tween-20 (PMT buffer), overnight at 4°C. Membranes were then washed 3 times with PMT buffer for 15 min before incubation with GAR-Po (Sigma-Aldrich) in PMT buffer for 45 min. Finally, membranes were washed 3 times with PBS containing 0.5% Tween-20. Peroxidase activity was detected using ECL chemiluminescence reagents (Amersham Biosciences, Buckinghamshire, UK) and revealed by exposure to X-ray. Protein bands were analyzed by densitometry using the Scion Image 4.0.2 Software (RRID: SCR_008673) ( www.scionorg.com).

### ROS measurement

The intracellular ROS level was assessed in VHSV-exposed RBCs using the cell-permeant 2',7'-dichlorodihydrofluorescein diacetate (DCFDA, Sigma-Aldrich). RBCs were exposed to VHSV at MOI 1, during 72 h, at 14°C. After that, RBCs were washed with RPMI and incubated with 20 μM DCFDA in RPMI, for 30 min at RT. Fluorescence intensity of 2′,7′-dichlorofluorescin was measured using POLARstar Omega microplate reader (BMG LABTECH, USA) at excitation 480 nm and emission 530 nm.

### Software and statistics

*Graphpad Prism 6* (RRID:SCR_002798, www.graphpad.com) was used for graphic representation and statistics calculation. Statistic tests and *P*-values associated with graphics are indicated in each assay. Flow cytometry data was processed and analyzed using *Flowing Software 2.5.1* ( www.flowingsoftware.com/) (RRID: SCR_015781).

### Ethics statement

All experimental protocols and methods of the experimental animals were reviewed and approved by the Animal Welfare Body and the Research Ethics Committee at the University Miguel Hernandez (approval number 2014.205.E.OEP; 2016.221.E.OEP) and by the competent authority of the Regional Ministry of Presidency and Agriculture, Fisheries, Food and Water supply (approval number 2014/VSC/PEA/00205). All methods were carried out in accordance with the Spanish Royal Decree RD 53/2013 and EU Directive 2010/63/EU for the protection of animals used for research experimentation and other scientific purposes.

## Results

### VHSV course of replication in rainbow trout RBCs

**Figure 1.**
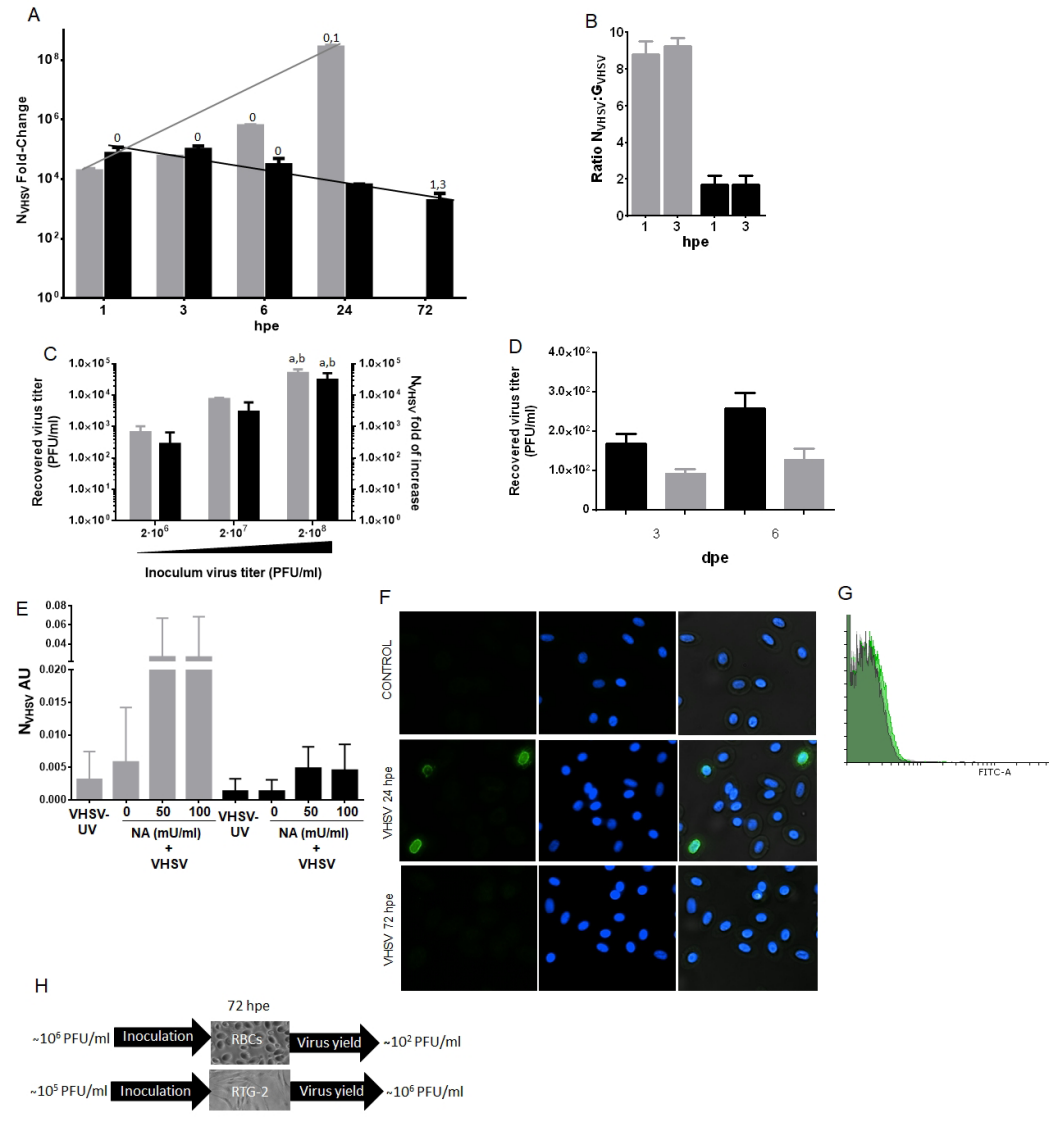
VHSV exposure and replication in rainbow trout RBCs. ( **A**) Time course of VHSV gene replication in rainbow trout RBCs and RTG-2 cell line. N gene of VHSV (N _VHSV_) expression profile was quantified by RT-qPCR at time 0, 1, 3, 6, 24 and 72 hours postexposure (hpe) to VHSV, in RBCs (black bars) and RTG-2 (grey bars), with a multiplicity of infection (MOI) of 1 at 14°C. Gene expression was normalized against eukaryotic 18S rRNA and *ef1α*, respectively for RBCs and RTG-2 cells, and relativized to control cells (non-exposed, time 0) (fold-change). Data represent the mean ± SD (n = 4 for RBCs and n=2 for RTG-2). ( **B**) Ratio of N _VHSV _and G _VHSV _genes expression by RT-qPCR at time 0, 1, and 3 hpe, relative to control cells (non-exposed, time 0), in RBCs (black bars) and RTG-2 (grey bars). Ratio was calculated as 2 ^−ΔΔCt^ N _VHSV_: 2 ^−ΔΔCt^ G _VHSV_. Gene expression was normalized against *ef1α*. Data represent the mean ± SD (n = 3 for RBCs and n=2 for RTG-2). ( **C**) Viral yield in VHSV-exposed rainbow trout RBCs. Viral titer (grey bars) (plaque forming units per millilitre, PFU/ml) and N _VHSV_ gene expression by RT-qPCR (black bars) of VHSV-exposed RBCs, with MOI 1, 10 and 100, respectively corresponding to inoculum virus titers 2·10 ^6 ^( **a**), 2·10 ^7^ ( **b**) and 2·10 ^8^ ( **c**) PFU/ml, 72 hpe, at 14°C. Gene expression was normalized against *ef1α.* Data represent the mean ± SD (n = 3 for viral titer and n=4 for N _VHSV_ gene expression). ( **D**) VHSV titers diminished in rainbow trout RBCs after NH _4_Cl treatment. VHSV titers obtained in VHSV-exposed RBCs at MOI 1, at 3 and 6 days postexposure (dpe), at 14°C, in the absence (black bars) or in the presence (grey bars) of NH _4_Cl. Data represent the mean ± SD (n = 4). ( **E**) Pre-treatment of RBCs with neuraminidase enhances early replication of VHSV. RBCs were inoculated with UV-inactivated or live VHSV, with a MOI of 1, at 14°C. Before infection, cells were pretreated with neuraminidase (NA) at 50 or 100 mU/ml during 30 minutes at 14°C. VHSV infectivity was quantified by N _VHSV_ gene expression analysis by RT-qPCR at 3 hpe (grey bars) and 72 hpe (black bars). Gene expression was normalized against 18S rRNA gene and represented as arbitrary units (AU). Data represent the mean ± SD (n = 4). ( **F**) Representative immunofluorescence of VHSV labelling in RBCs exposed to VHSV (MOI 100, 24 and 72 hpe, 14°C) stained from left to right with anti-N _VHSV_ 2C9 (FITC), DAPI for nuclei stained and merged (IF representative of 32 images). ( **G**) Representative flow cytometry overlay histograms showing untreated RBCs (grey filled histogram), VHSV-exposed RBCs with a MOI 100, at 14°C, 24 hpe (green filled histogram) and 72 hpe (black filled histogram). ( **H**) Schematic representation of the VHSV infectivity in RBCs and RTG-2 cells, indicating the virus inoculation titer and recovered virus yield after 72 hpe in each cell line. Kruskal-Wallis Test with Dunn´s Multiple Comparison post-hoc test was performed for statistical analysis among all conditions. Values over the bars denote pairwise significant differences with the value-indicated time point or condition ( *P*-value < 0.05).

For this analysis we first purified RBCs (oval nucleated cells) to 99.9% (as evaluated by optical microscopy) and then exposed the purified RBCs to VHSV, for different times, to monitor replication of VHSV in rainbow trout RBCs. For that, time course expression of N gene of VHSV (N _VHSV_) was measured by RT-qPCR with cDNA performed with random hexamer primers (to target total RNA). Expression of N _VHSV_ gene was significantly upregulated at 3 hpe. However, it drastically decreased from 6 to 72 hpe, indicating that VHSV could replicate at early times postexposure, at the same levels as VHSV susceptible rainbow trout cell line RTG-2. However, viral replication was halted in RBCs at later stages of infection, in contrast to RTG-2 ( Figure 1A). Besides, cDNA synthesis was also performed with oligo(dT) primers to target N _VHSV_ mRNA expression in VHSV-exposed RBCs and the result was consistent with total RNA expression ( Figure S3). On the other hand, after VHSV enters the cell, the first gene that starts to transcribe is N _VHSV _gene, since it is the closest to the 3’ transcriptional start, and the more distal, excluding the polymerase, is the G glycoprotein gene (G _VHSV_) gene. Therefore, under a normal transcription scenario a high ratio between the N _VHSV_ and G _VHSV_ viral genes transcripts is to be expected, taking into account the attenuation phenomenon found in rhabdoviruses ^47, 48^. However, a ratio of 2 was observed in RBCs, compared to the ratio of 8 found in RTG-2 cells, at 1 and 3 hpe ( Figure 1B).

Also, RBCs were exposed to different VHSV multiplicities of infection (MOI). Initial VHSV inoculum titer declined ~4-logs after 3 days of incubation at the indicated MOI assayed (1, 10 or 100, respectively corresponding to inoculum virus titers 2·10 ^6^, 2·10 ^7^, 2·10 ^8 ^PFU/ml) ( Figure 1C), in contrast to the usual 1-log titer increment in RTG-2 cells infected in the same conditions ( Figure 1H). Later on, RBCs showed only a minor and statistically non-significant ~1-fold increment of VHSV titer as time of infection increased from 3 to 6 days ( Figure 1D), showing that low VHSV titers are maintained in the cell after 72 hpe. These low VHSV titers were due to true VHSV internalization and not to residual VHSV binding, since they were diminished with NH _4_Cl treatment, a characteristic of rhabdovirus infections ( Figure 1D). NH _4_Cl acts as a lysosomotropic drug, blocking endosomal acidification and inhibiting rhabdoviral cytoplasmic entrance steps including those of VHSV ^33^.

In order to increase the amount of VHSV inside rainbow trout RBCs, RBCs were pre-treated with neuraminidase (NA) and then exposed to UV-inactivated or live VHSV. NA has been shown to enhance rhabdovirus infection in NA pre-treated cells by favoring interaction with cellular membranes ^49^. As a result, VHSV RNA inside RBCs was increased about ten times at 3 hpe in live VHSV-exposed RBCs, in comparison with UV-inactivated VHSV-exposed RBCs. However, seventy-two hpe the VHSV RNA drastically decreased to almost disappear, as indicated by N _VHSV_ RT-qPCR ( Figure 1E).

Besides, N _VHSV_ protein (2C9 antibody) was detected in RBCs exposed to VHSV MOI 100, at 24 hpe, but not at 72 hpe. IF images ( Figure 1F) showed an intracellular stain, mainly in nuclear and perinuclear regions. FC histogram ( Figure 1G) showed a slight increment of VHSV N protein in VHSV-exposed RBCs, at 24 hpe, but not at 72 hpe. VHSV could not be detected by IF or FC in RBCs exposed to lower MOIs.

### Antiviral transcriptional immune responses in rainbow trout RBCs exposed to VHSV *in vitro*

**Figure 2.**
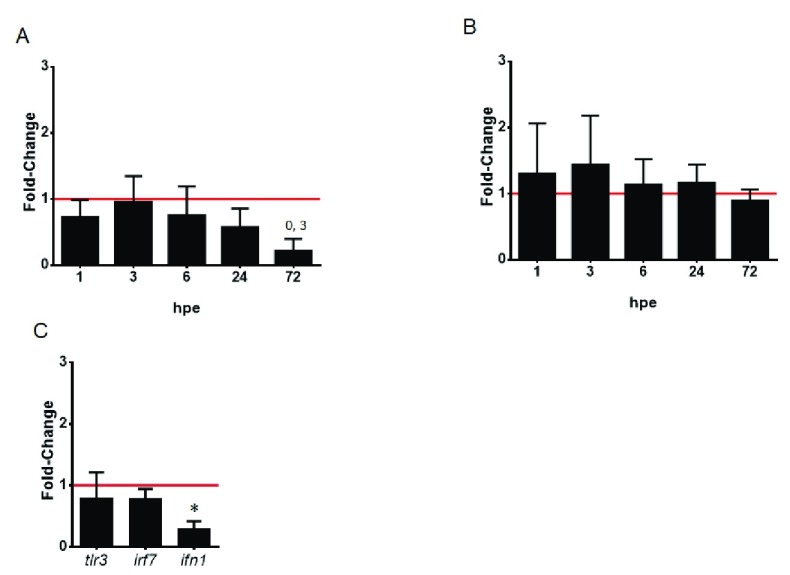
Interferon signaling in VHSV-exposed rainbow trout RBCs. Time course of interferon-inducible antiviral genes *mx* ( **A**) and *pkr* ( **B**). RBCs were exposed to VHSV with a multiplicity of infection (MOI) of 1 at 14°C, and *mx1-3* and *pkr* genes expression was quantified by RT-qPCR at time 0, 1, 3, 6, 24, 72 hours postexposure (hpe). Data is displayed as mean ± SD (n = 3). Kruskal-Wallis Test with Dunn´s Multiple Comparison post-hoc test was performed among all conditions. ( **C**) Interferon signaling at early time postexposure. RBCs were exposed to VHSV with a MOI of 1 at 14°C, and *tlr3*, *irf7* and *ifn1* gene expression profiles were quantified by RT-qPCR at time 0, and 3 hpe. Data is displayed mean ± SD (n = 3). Mann Whitney Test was performed for statistical analysis between the VHSV-exposed and control cells (non-exposed, time 0, red line). Gene expression was normalized against eukaryotic 18S rRNA for *mx*, *tlr3*, *irf7* and *ifn1* genes and *ef1α* for *pkr* gene, and relativized to control cells (fold-change). Asterisk denote statistically significant differences between VHSV-exposed and control cells ( *P*-value < 0.05).

We next investigated whether rainbow trout RBCs exposed to VHSV could be capable of generating immune responses *in vitro*, by means of examining the differential expression profile of some genes characteristic of fish antiviral response. First, a time course monitoring of the expression of interferon-inducible *mx* and *pkr* genes was carried out at different time postexposure. The results showed that *mx* and *pkr* genes exhibited the same increment peak at 3 hpe and a tendency to downregulation from 6 to 72 hpe, in parallel to N _VHSV_ gene transcription levels tendency ( Figure 2A and B, and Figure 1A). The expression of *mx* and *pkr* genes did not change over the time-course in control cells ( Figure S4). On the other hand, at 3 hpe, *ifn1* gene expression already exhibited a statistically significant downregulation ( Figure 2C), and a slight downregulation for *tlr3* and *irf7* genes.

### Antiviral immune protein responses in RBCs exposed to VHSV *in vitro*

**Figure 3.**
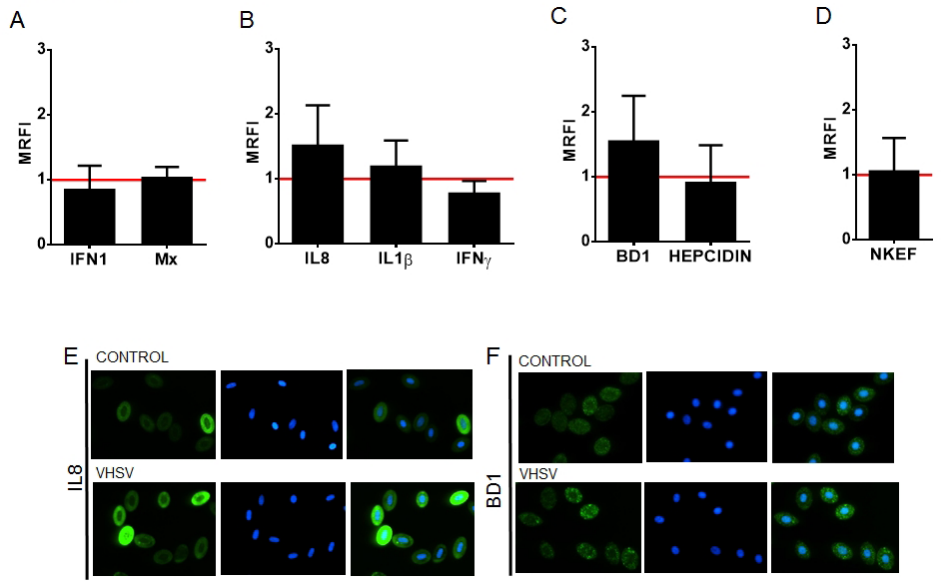
Immune protein responses of VHSV-exposed RBCs. Relative immune protein expression levels, ( **A**) interferon pathway related proteins (IFN1 and Mx), ( **B**) cytokines (IL8, IL1β and IFNγ), ( **C**) antimicrobial peptides (BD1 and Hepcidin) and ( **D**) antioxidant protein NKEF, measured by flow cytometry and calculated by the formula MRFI (Mean Relative Fluorescence Intensity) = fluorescence in VHSV-exposed RBCs / fluorescence in non-exposed RBCs, at multiplicity of infection (MOI) 1, 72 hours postexposure (hpe), at 14°C, relative to control cells (non-exposed, red line). Data is displayed as mean ± SD (n=5). Mann Whitney Test was performed for statistical analysis between the VHSV-exposed cells and control cells. Representative immunofluorescences of control and VHSV-exposed RBCs stained with anti-IL8 (IF representative of 44 images) ( **E**) and anti-BD1 (IF representative of 46 images) ( **F**) (FITC) and DAPI for nuclei stain.

Changes in RBCs immune protein response induced by VHSV exposure were assessed using specific antibodies. VHSV-exposed RBCs showed only an increment in protein levels of chemokine IL8 ( Figure 3B and E, Figure S5A) and antimicrobial peptide BD1 ( Figure 3C and F, Figure S5B), verified by means of FC and IF. Mx and IFN1 protein levels, according to the RT-qPCR results, did not change or downregulate, respectively ( Figure 3A). Cytokines IL1β, IFNγ ( Figure 3B), antimicrobial peptide Hepcidin ( Figure 3C) and natural killer enhancing factor (NKEF) ( Figure 3D) did not show regulation at 72 hpe.

### Interferon crosstalk between RBCs and spleen stromal TSS cell line

**Figure 4.**
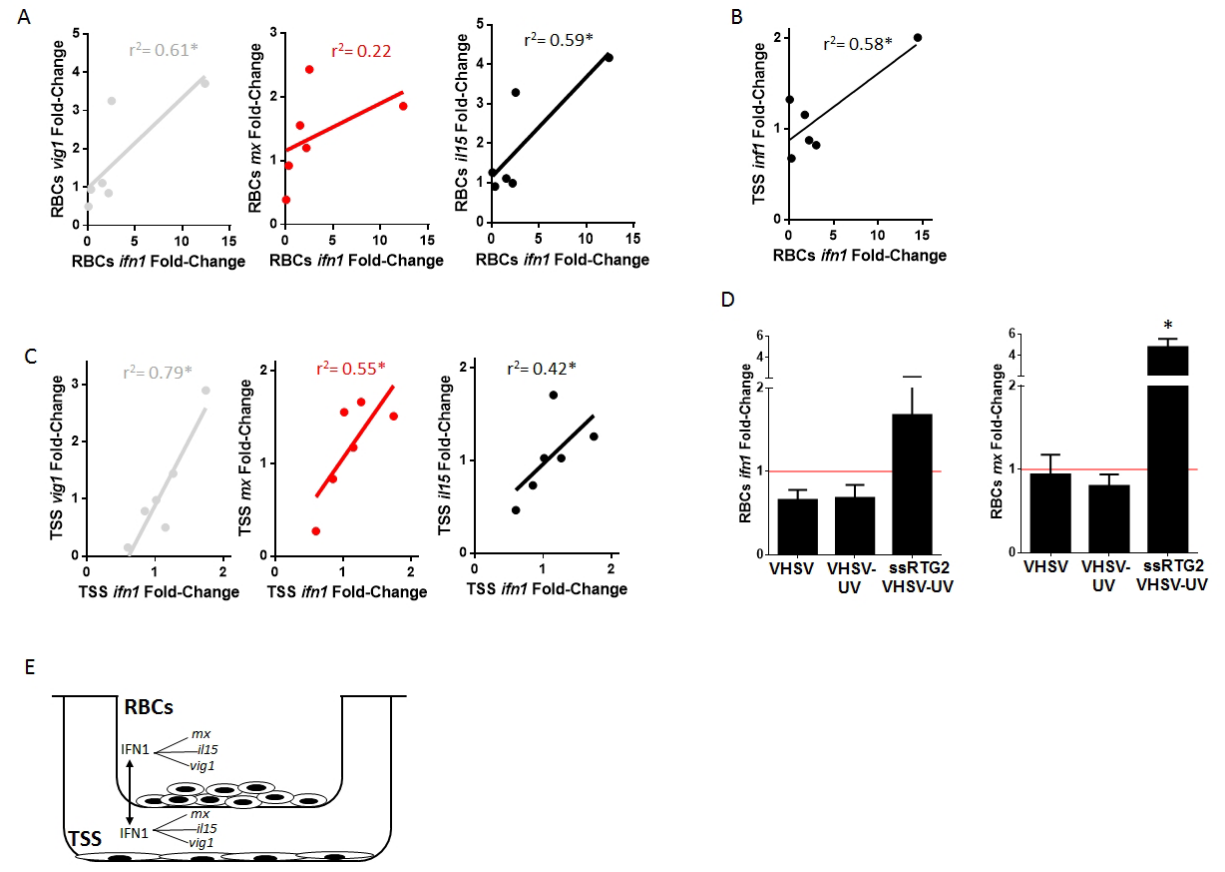
Crosstalk between rainbow trout RBCs and spleen stromal cell line TSS. Rainbow trout RBCs, control (non-exposed) and exposed to UV-inactivated VHSV, with multiplicity of infection (MOI) 1, were posteriorly co-cultured with TSS cell line, at 14°C, and *ifn1*, *mx*, *vig1* and *il15* gene expression profiles were quantified by RT-qPCR at 24 hours postexposure (hpe) for RBCs and TSS. ( **A**) Linear regression between *ifn1* and interferon stimulated genes *vig1*, *mx*, and *il15* gene expression profiles in RBCs. ( **B**) Linear regression between RBCs and TSS *ifn1* gene expression profile. ( **C**) Linear regression between *ifn1* and *vig1*, *mx*, and *il15* gene expression profiles in TSS. Gene expression was normalized against eukaryotic 18S rRNA and relativized to control cells (red line) (fold-change). Data is displayed as a linear regression line, with individual dots, between indicated cell lines and expressed genes (r ^2^: coefficient of determination, asterisk denote statistical significance, *P*-value < 0.05) (n = 6). ( **D**) Rainbow trout RBCs exposed to VHSV, UV-inactivated VHSV (VHSV-UV) (MOI 1, 14ºC, 24h) or treated with conditioned medium from RTG-2 cells pre-treated with VHSV-UV, for 24h at 14ºC. RBCs *ifn1* and *mx* gene expression profiles were quantified by RT-qPCR. Gene expression was normalized against eukaryotic 18S rRNA and relativized to control cells (RBCs incubated with conditioned medium from untreated RTG-2 cells, red line) (fold-change). Data represent the mean ± SD (n = 4). Kruskal-Wallis Test with Dunn´s Multiple Comparison post-hoc test was performed among all conditions. Asterisk denote significant differences with the indicated condition and control cells ( *P*-value < 0.05). ( **E**) Schematic representation of RBCs and TSS co-culture assay and analysis.

Rainbow trout spleen is an active hematopoietic organ ^50^, and it is composed of various cell types, such as red blood cells, leukocytes and reticular or stromal cells ^51^. It has been demonstrated that cytokines and soluble factors produced by stromal cells are required for rainbow trout blood cells development in spleen or head kidney ^52^. In this regard, we wanted to evaluate the paracrine effects of the cytokines produced by VHSV stimulated RBCs over the stromal cell line from rainbow trout spleen, TSS ^29^. For that, rainbow trout RBCs stimulated with VHSV UV-inactivated were co-cultured with TSS cell line, using a Transwell system to test whether a cross-stimulation mediated by soluble molecules was involved. Gene expression profiles for *ifn1*, and interferon stimulated genes (ISGs) *mx*, viral inducible gene *vig1*, and interleukin *il15* genes were examined for each cell line 24 hours post co-culture ( Figure 4E). Linear regression analysis of RBCs *ifn1* gene expression with their respective *mx, vig1* and *il15* genes showed a significant correlation between *ifn1* and *vig1* and *il15*, but not with *mx* gene ( Figure 4A). *ifn1* gene expression from RBCs and TSS cells also showed a significant correlation ( Figure 4B). TSS cells showed significant correlation between *ifn1* and *mx*, *vig1* and *il15* ( Figure 4C). The results demonstrated an IFN crosstalk between stimulated RBCs and TSS cells. Besides, this IFN crosstalk was also observed when RBCs were incubated with conditioned medium from RTG-2 cells previously treated with UV-inactivated VHSV, since we could observe an increment in *ifn1* and *mx* genes expression, in contrast to *ifn1* or *mx* downregulation when RBCs were directly exposed to VHSV ( Figure 4D).

### iTRAQ protein profile of VHSV-exposed RBCs

**Figure 5.**
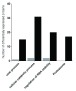
Gene ontology (GO) analysis of iTRAQ-based differentially expressed proteins in VHSV-exposed rainbow trout RBCs. RBCs were exposed to VHSV with a multiplicity of infection (MOI) of 1 at 14°C, and protein quantified at 72 hpe. Proteins were classified into five specific GO-Biological Process categories indicated in the x-axis. The y-axis indicates the number of proteins in each category. Grey bars indicate upregulated proteins and black bars down-regulated proteins.

**Figure 6.**
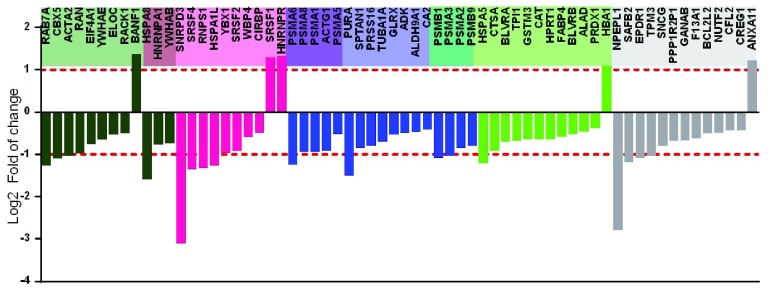
iTRAQ-based quantitative protein expression profile of VHSV-exposed rainbow trout RBCs. Bar plot of statistically significant differentially expressed proteins in VHSV-exposed RBCs (MOI 1, 14°C, 72 hpe) compared to control cells (non-exposed) ( *P*-value < 0.05, FDR *q*-value < 0.05). Functional categories are labelled as follows: Blue = proteasome, pink = regulation of RNA stability, light green = cellular catabolic process, dark green= viral process, grey = proteins not associated to any function.

The iTRAQ data showed a total of 9246 MS/MS Spectra, 2639 unique peptides with peptide-level FDR<0.01 and 872 inferred proteins common in all samples. Significant up/down regulations between samples were determined by a log2FoldChange)>1 with a *q*-value<0.05. In total, 64 proteins were significantly up or down-regulated during VHSV exposure ( Figure 5). Specifically, 59 proteins were downregulated and only 5 proteins were upregulated during VHSV exposure. Cytoscape functional annotation was used to investigate underlying biologically functional differences that may be related to VHSV exposure. The results showed four strongly represented networks of interest (mRNA stability, proteasome, viral process and cellular catabolic processes) ( Figure 5 and Figure S6). Among the 59 down-regulated proteins ( Figure 6, Table S1), the top-score network was mRNA stability, being SNRPD3 (Small nuclear ribonucleoprotein D3 polypeptide) the most down-regulated protein with ~ -3 log2FoldChange. This protein is a core component of spliceosomal small nuclear ribonucleoproteins (snRNPs), the building blocks of the spliceosome, and therefore, it plays an important role in the splicing of cellular pre-mRNAs. Other proteins related to splicing processes were also highly downregulated (-2>log2FoldChange>-1), such as SRSF4 (Serine/arginine-rich splicing factor 4), which plays a role in alternative splice site selection during pre-mRNA splicing, RNPS1 (RNA binding protein S1, serine-rich domain), which is part of pre- and post-splicing multiprotein messenger ribonucleoprotein (mRNP) complexes. Apart from that, several heat shock chaperones were also downregulated (-2>log2FoldChange>-1), such as HSPA1L (Heat shock 70kDa protein 1-like) and HSPA5 (Heat shock 70kDa protein 5) both involved in the correct folding of proteins and degradation of misfolded proteins, and HSPA8 (Heat shock 70kDa protein 8), which may have a scaffolding role in the spliceosome assembly. Besides, another protein highly downregulated was NPEPL1 (Aminopeptidase-like 1), a novel protein which has been implicated in HIV replication ^53^.

On the other hand, among the five upregulated proteins ( Figure 6, Table S1), BANF1 (Barrier to Autointegration factor 1) has been directly implicated in viral processes and plays fundamental role in nuclear assembly, chromatin organization and gene expression. Besides, HNRNPR (Heterogeneous nuclear ribonucleoprotein R) plays an important role in processing precursor mRNA in the nucleus, and SRSF1 (Serine/arginine-rich splicing factor 1) is also implicated in mRNA splicing, via spliceosome.

**Figure 7.**
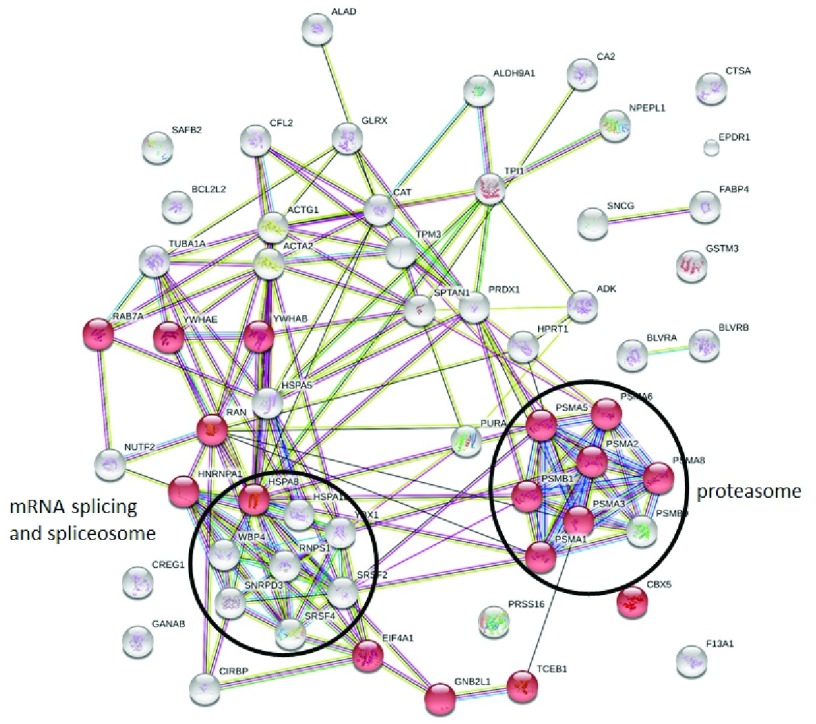
Constructed protein-protein interaction of differentially downregulated proteins (DDPs) predicted using STRING software. Nodes represent DDPs and edges the interactions between two proteins. The colour of the edge indicates the interaction score (edge score). Red nodes highlight DDPs functionally annotated in viral processes.

The 59 downregulated proteins were analyzed using STRING v10.5 (RRID:SCR_005223, http://string.embl.de/) ^54^ with a medium confidence score threshold of 0.4. An interactome network was built for these set of proteins to find out protein-protein interaction and predict functional associations. We found that proteins within spliceosome and proteasome networks interacted with each other as well as with their partners. We also found that 17 proteins were involved in viral process category and that most of them interacted with each other as well as with their partners ( Figure 7).

### Phosphorylation of eIF2α in VHSV-exposed RBCs

**Figure 8.**
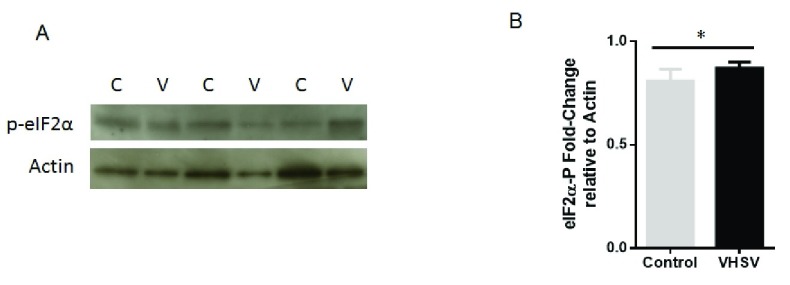
Phosphorylation of translation initiation factor eIF2α in VHSV-exposed rainbow trout RBCs. ( **A**) Representative western blot of eIF2α phosphorylation (eIF2α-P) in VHSV-exposed (V) (MOI 1, 14°C, 72 hpe) and control RBCs (C) (non-exposed). ( **B**) Bar plot of eIF2α-P protein content of stained bands estimated by densitometry, relative to α-Actin. Mann Whitney Test was performed for statistical analysis between VHSV-exposed cells and control cells. Asterisk denote statistically significant differences ( *P*-value < 0.05).

Since a global protein downregulation was observed in VHSV-exposed RBCs, we further investigated whether this phenomena could be due to the phosphorylation of the α-subunit of translational initiation factor 2 (eIF2α), a recognized key mechanism of global inhibition of translational initiation. For that, phosphorylation of eIF2α (eIF2α-P) was evaluated in VHSV-exposed RBCs compared to control cells by western blot ( Figure 8A and B). The results showed a small upregulation of eIF2α-P in VHSV-exposed RBCs.

**Figure 9.**
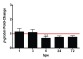
*β-globin* gene expression time-course in VHSV-exposed rainbow trout RBCs. RBCs were exposed to VHSV with a multiplicity of infection (MOI) of 1 at 14°C. Gene expression was quantified by RT-qPCR at time 0, 1, 3, 6, 24, 72 hours postexposure (hpe). Gene expression was normalized against eukaryotic 18S rRNA and relativized to control cells (non-exposed, time 0, red line) (fold-change). Data is displayed as mean ± SD (n = 3). Kruskal-Wallis Test with Dunn´s Multiple Comparison post-hoc test was performed among all conditions. Values denote pairwise significant differences with the value-indicated condition ( *P*-value < 0.05).

Four eIF2α kinases have been identified to inhibit protein synthesis by phosphorylation of eIF2α: double-stranded RNA-dependent eIF2α kinase (PKR), mammalian orthologue of the yeast GCN2 protein kinase, endoplasmic reticulum (ER) resident kinase (PERK) and heme-regulated eIF2α kinase (HRI) ^55^. HRI, which was first discovered in reticulocytes under conditions of iron and heme deficiencies ^56, 57^, was later known to regulate the synthesis of both α- and β-globins in RBCs and erythroid cells by phosphorylation of eIF2α ^58^, and therefore inhibiting protein synthesis. Besides, heme is also known to regulate the transcription of globin genes through its binding to transcriptional factor Bach1 ^59^. Taking this fact into account, we explored RBCs *β-globin* gene expression during the course of VHSV exposure and the results showed that *β-globin* gene was downregulated after 6 hpe ( Figure 9), therefore suggesting an activation/phosphorylation of HRI and consequent phosphorylation of eIF2α and protein inhibition.

### Oxidative stress and antioxidant response in VHSV-exposed RBCs

**Figure 10.**
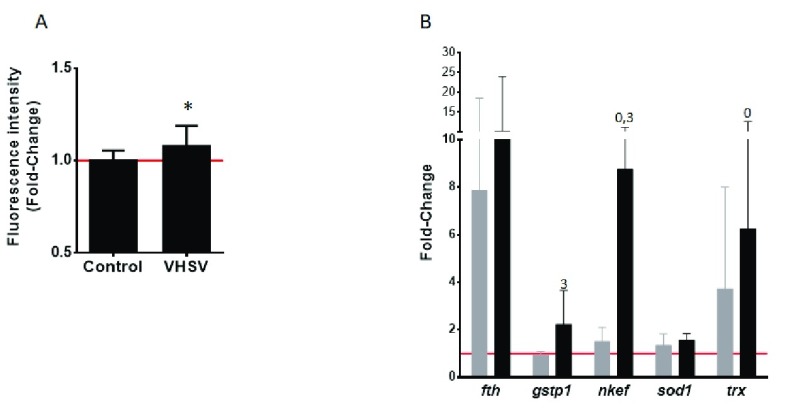
Effect of VHSV on ROS intracellular production and antioxidant enzymes gene expression in rainbow trout RBCs. RBCs were exposed to VHSV with a multiplicity of infection (MOI) of 1 at 14°C, ( **A**) DCFDA (2′,7′-Dichlorofluorescin diacetate) fluorescence intensity of VHSV-exposed RBCs relative to control cells (non-exposed), 72 hours postexposure (hpe). ( **B**) Antioxidant genes ( *fth*: ferritin, *gstp1*: glutathione S-transferase P, *nkef*: natural killer enhancement factor-like protein, *sod1*: superoxide dismutase [Cu-Zn], *trx*: thioredoxin) gene expression quantified by RT-qPCR at 3 hpe (grey bars) and 72 hpe (black bars). Gene expression was normalized against eukaryotic 18S rRNA and relativized to control cells (time 0, red line) (fold-change). Data is displayed as mean ± SD (n = 3). Kruskal-Wallis Test with Dunn´s Multiple Comparison post-hoc test was performed among all conditions. Values denote pairwise significant differences ( *P*-value < 0.05) with the value-indicated condition.

Oxidative stress is known to be induced by viral infections, being one of the major pathogenic mechanisms by altering the balance of intracellular redox ^60^. On the other hand, oxidative stress is known to activate HRI, which in turn phosphorylates eIF2α and inhibits protein translation. In order to evaluate the oxidative stress induced in VHSV-exposed RBCs as possible causative mechanism for the proteome downregulation found in our study, we examined, at 72 hpe, the ROS intracellular production by means of DCFDA (2′,7′-Dichlorofluorescin diacetate) fluorescence intensity. The results showed that VHSV-exposed RBCs significantly augmented DCFDA fluorescent intensity 72 hpe ( Figure 10A), therefore VHSV halted infection in RBCs generated oxidative stress in rainbow trout RBCs. Besides, in order to evaluate the capability of RBCs to respond to oxidative stress, antioxidant response of VHSV-exposed RBCs was evaluated examining transcript levels of antioxidant genes *fth* (ferritin), *gstp1* (glutathione S-transferase P), *nkef* (natural killer enhancement factor-like protein), *sod1* (superoxide dismutase [Cu-Zn]) and *trx* (thioredoxin). The results depicted an increment in transcript levels of *fth*, *gstp1*, *nkef* and *trx* ( Figure 10B) as the time of exposure increased from 3 to 72 hours, demonstrating the capability of rainbow trout RBCs to counteract oxidative stress.

Excel file containing qPCR data. Each sheet contains the raw Ct values for the indicated figure numbers, organized by samples (rows) and genes (columns) Click here for additional data file. Copyright: © 2018 Nombela I et al. 2018 Data associated with the article are available under the terms of the Creative Commons Zero "No rights reserved" data waiver (CC0 1.0 Public domain dedication).

Excel file containing the virus titration data. Each sheet contains the virus titer (PFU/mL) results of the indicated figure number Click here for additional data file. Copyright: © 2018 Nombela I et al. 2018 Data associated with the article are available under the terms of the Creative Commons Zero "No rights reserved" data waiver (CC0 1.0 Public domain dedication).

Flow cytometry data. Each folder contains the Flow Cytometry Standard (.fcs) format files. Source data files are organized by figure number, and then by antibody, sample number and condition Click here for additional data file. Copyright: © 2018 Nombela I et al. 2018 Data associated with the article are available under the terms of the Creative Commons Zero "No rights reserved" data waiver (CC0 1.0 Public domain dedication).

Excel file containing the computed peptide spectrum match (PSM) raw data, and the spectra recovered in the iTRAQ 4-plex analysis Click here for additional data file. Copyright: © 2018 Nombela I et al. 2018 Data associated with the article are available under the terms of the Creative Commons Zero "No rights reserved" data waiver (CC0 1.0 Public domain dedication).

Excel file containing the iTRAQ 4-plex quantitative analysis raw data Click here for additional data file. Copyright: © 2018 Nombela I et al. 2018 Data associated with the article are available under the terms of the Creative Commons Zero "No rights reserved" data waiver (CC0 1.0 Public domain dedication).

Excel file containing the densitometry raw data of eIF2α-P and α-Actin western blots. Related uncropped blots are included Click here for additional data file. Copyright: © 2018 Nombela I et al. 2018 Data associated with the article are available under the terms of the Creative Commons Zero "No rights reserved" data waiver (CC0 1.0 Public domain dedication).

Excel file containing DCFDA absorbance raw data Click here for additional data file. Copyright: © 2018 Nombela I et al. 2018 Data associated with the article are available under the terms of the Creative Commons Zero "No rights reserved" data waiver (CC0 1.0 Public domain dedication).

## Discussion

Most viral infections release their progeny to the outside of the cells (productive infections). However, viral infections can be also non-productive in non-permissive cells (also called abortive). Viral abortive infections occur when a virus enters a host-cell, then some or all viral components are synthesized but finally no infective viruses are released ^61^. This situation may result from an infection with defective viruses or because the host cell is non-permissive and inhibits replication of a particular virus. Our results are consistent with VHSV binding and internalization in rainbow trout RBCs. Internalization of VHSV occurs via fibronectin-integrin receptors in the host cell ^12^. Integrin proteins expression has been found in human red blood cells ^62^, however, it is unknown whether non-mammalian nucleated RBCs express integrins. VHSV internalization is followed by viral genes transcription at early times of viral exposure and posterior quasi-inhibition inside rainbow trout RBCs. In this sense, rainbow trout RBCs could be classified as a non-permissive cell for VHSV replication, in contrast to other rainbow trout cells or tissues where VHSV is productive, such as RTG-2 cells ^63, 64^, fin cells ^65^ or stroma ^66^. Therefore, from our results, VHSV infection could be classified as halted in rainbow trout RBCs, since it enters the cell, but does not replicate at the levels comparable to the ~100-fold increase in titre of PRV and ISAV infections in salmon RBCs ^5, 7^. In fact, an apparent inhibition of the early viral genes transcription seemed to occur since N _VHSV_: G _VHSV_ viral genes transcripts ratio was very low, and therefore did not follow the attenuation phenomenon found in rhabdoviruses ^47^. However, strikingly, even though recovered VHSV titer in the RBCs supernatant was very low at 3 and 6 dpe, at 40 dpe almost the same virus titer could be recovered from RBCs supernatant (data not shown), suggesting an *ex vivo* persistence of VHSV inside RBCs.

In the literature, innate immune responses have been associated with viral abortive infections, including rhabdoviruses. Pham *et al*. ^67^ speculated that the cause of aborted VHSV infection in rainbow trout macrophage cell line (RTS-11) could be the constitutive expression and/or upregulation of *mx* genes. The abortive infection of snakehead fish vesiculovirus (SHVV) in zebrafish embryonic fibroblast cell line (ZF4) was associated with activation of Retinoic acid-Inducible Gene I (RIG-I)-like receptors and interferon pathway by viral replicative intermediates ^68^. Similarly, in mammals, Pfefferkorn *et al.* ^69^ demonstrated that abortive viral infection of astrocytes by rabies virus (RABV) and vesicular stomatitis virus (VSV) triggered a pattern recognition receptor signaling which resulted in the secretion of IFN-β. On the other hand, it has been also described that alveolar macrophages are able to restrict respiratory syncytial virus (RSV) replication even in the absence of type I IFNs (IFN1) ^70^. In this sense, VHSV halted infection in rainbow trout RBCs did not seem to be related to IFN1 or IFN1-inducible genes, since *inf1*, *mx* and *pkr* genes as well as Mx and IFN1 proteins appeared poorly modulated or downregulated during VHSV exposure, in contrary to the 8-fold increase in ISAV productive infection in salmon RBCs ^7^, the 50-fold increases in PRV productive infection in salmon RBCs ^5^ or the 50-fold increases in IPNV non-productive infection in rainbow trout RBCs ^71^. Alternatively, high levels of constitutive Mx protein expression might have prevented its further increase in VHSV-exposed RBCs, like it is the case of the rainbow trout monocyte-macrophage RTS-11 cell line ^72^. On the other hand, several cell mechanisms have been reported to suppress IFN1-mediated responses, which include downregulation of cell surface IFNα receptor (IFNAR) expression, induction of negative regulators (such as suppressor of cytokine signalling (SOCS) proteins and ubiquitin carboxy-terminal hydrolase 18 (USP18)), as part of a negative feedback loop to limit the extent and duration of IFN1 responses ^73^. Separately, a putative antagonistic effect of VHSV virus on Mx induction has been previously reported ^74, 75^. From our results, in VHSV-exposed RBCs, *mx* gene poor induction or slight downregulation could be probably supported by the existence of a VHSV antagonistic effect against RBCs IFN response. To further clarify whether a viral antagonistic effect or a feedback loop of IFN1 and/or IFN1-inducible genes induction is related to or responsible for aborting or halting viral infections in rainbow trout RBCs remains to be studied, and are part of our ongoing research.

Separately, although the IFN levels were low, our results demonstrated the paracrine IFN crosstalk between RBCs, stimulated with UV-inactivated VHSV, and spleen stromal TSS cell line. TSS cell line has been described to resemble the immune responses observed in cultures of head kidney macrophages ^76^. Also, it has been demonstrated the ability of TSS to positively respond to conditioned supernatants from head kidney macrophage cultures exposed to poly I:C ^76^. As well, after exposure to poly I:C, TSS produced a high upregulation of the Mx-1 gene ^77^. Our results showed the correlated *ifn1* regulation in both cell lines, as well as the correlative regulation of interferon-inducible *mx* gene in TSS, the regulation of *il15*, an interleukin that can activate antiviral responses via an interferon-dependent mechanism ^78^, and the regulation of VHSV-inducible *vig1*, a gene induced by VHSV as well as by interferon ^79^. Separately, we observed that conditioned medium from RTG-2 cells previously treated with UV-inactivated VHSV could induce an increment in *ifn1* and *mx* genes expression in RBCs, in contrast to *ifn1* or *mx* observed downregulation when RBCs were directly exposed to VHSV. Therefore, this crosstalk observations demonstrated the capacity of rainbow trout RBCs to exert a paracrine molecular antiviral communication with other cells with capacity to generate an immune response, as it is the case of the TSS cell line ^77^ or RTG-2 cells ^15^. More extended research is need to further identify the molecules involved in this crosstalk.

On the other hand, other immune proteins, such as BD1, IL1β and IL8, known to be involved in antiviral immunity, which were upregulated in VHSV- exposed RBCs, appeared to be part of the antiviral immune response of rainbow trout RBCs and could be implicated in the halted viral replication inside RBCs.

To further investigate the mechanisms implicated in the immune response of rainbow trout RBCs to VHSV, the comprehensive analysis of differentially expressed proteins, obtained by means of iTRAQ proteome profiling, revealed the regulation of two typical mechanisms for viral subversive strategies: regulation of spliceosome, or splicing hijacking, and host-cell shut-off. However, even though these strategies usually lead to viral augmented replication and cell death, in the case of VHSV-exposed RBCs this is not observed. Therefore, how these strategies or another strategies contribute to halting viral replication yet remains elusive. Future research could be directed to investigate the role/implication of small nuclear ribonucleoprotein SNRPD3, aminopeptidase NPEPL1, serine/arginine-rich splicing factor SRSF1 and heterogeneous nuclear ribonucleoprotein HNRNPR, in the response of RBCs against VHSV replication, since these proteins were the more regulated ones and they have been shown to be implicated in HIV replication ^53, 80– 82^).

On the other hand, inhibition of both host and viral translation has been shown during infection with the prototype rhabdovirus vesicular stomatitis virus (VSV) ^83^. During VSV infection, there is a rapid inhibition of host mRNA translation early after infection, followed by a later inhibition of viral mRNA translation, which has been associated to eIF2α phosphorylation ^84^. Our results showed a slight increment in eIF2α phosphorylation in VHSV-exposed RBCs, indicating that this mechanism could be implicated in the inhibition of VHSV replication in rainbow trout RBCs. In this context, HRI, heme-regulated eIF2α kinase, is one of the four kinases identified to inhibit protein synthesis by means of eIF2α phosphorylation. HRI is predominantly expressed in reticulocytes and erythroid precursors ^56, 57^, and it is known to regulate the synthesis of both α- and β-globins in RBCs and erythroid cells by phosphorylation of eIF2α ^58^. Moreover, heme, the prosthetic group of hemoglobin, is known to inhibit eIF2α and therefore the transcription of globin genes through its binding to transcriptional factor Bach1. From our results, a decrease in *β-globin* gene transcripts levels during the course of viral exposure, accompanied with the observed phosphorylation of eIF2α, could suggest a possible heme regulation mechanism of eIF2 pathway in response to VHSV exposure in rainbow trout RBCs. The mechanism by which heme is altered in rainbow trout RBCs during VHSV exposure remains to be investigated.

An interesting mechanism found in rainbow trout RBCs in response to VHSV was the implication of protective antioxidant enzymes genes *gstp1*, *nkef* and *trx* in the defense of RBCs against the induction of ROS after VHSV exposure, since as the course of virus exposure increased, ROS slightly augmented in parallel to transcript levels of these enzymes. It is known that ROS plays an important role in cell signalling and immunomodulation among others ^85, 86^ as well as performing antimicrobial actions against pathogens ^87^. However, oxidative stress due to imbalance in the production/elimination of ROS can have cytotoxic effects ^88^. ROS scavengers are the major defense against oxidative stress produced in the cells ^88^. These systems are known to contribute not only to repair the oxidative damage maintaining redox homeostasis, but also to the overall response of the cell to ROS by acting as oxidative sensors in signal transduction pathways ^89^. However, although it has been said that ROS production contributes to eliminate pathogens, nowadays it is becoming evident that viruses, bacteria, and protozoans ROS induction can also promote pathogen burden ^90^. In this regard, in relation to the implication of antioxidants activity against viral replication, it has been also described that antioxidants can suppress virus-induced oxidative stress and reduce RNA virus production ^91^. GSTP1, NKEF and TRX are known antioxidant enzymes with implication and up-regulation in RNA-virus infections ^92^ and rhabdovirus infections ^24, 93^. However, whether these enzymes may contribute to halting or reducing viral replication remains to be studied. On the other hand, mammals’ RBCs have an extensive array of antioxidant enzymes to counteract oxidative stress and maintain redox homeostasis and RBCs survival ^94^. However, to our knowledge this is the first report that implicates nucleated RBCs ROS scavengers in the antiviral immune response. Separately, these antioxidant enzymes are known NF-κβ antioxidant targets in response to inflammation stimulus (reviewed in Morgan and Liu, 2011 ^89^) and ROS can be sometimes produced in response to cytokines. Since NF-κβ appeared slightly activated in VHSV-exposed RBCs ( Figure S7A and B), it is suggested that the cytokine response generated after VHSV exposure in rainbow trout RBCs would induce ROS production, and in turn this would modulate the NF-κβ response and NF-κβ target genes could attenuate ROS to promote RBCs survival. Apart from the observation of NF-κβ translocation to the nucleus in some of the RBCs, it is noteworthy that it is always accompanied by an increase in the protein levels of the p65 NF-κβ subunit in the cytoplasm. This phenomenon has been also observed in human foreskin fibroblasts during HCMV infection, where an increase in p65 mRNA levels correlated with the sustained increase in NF-kB activity during the course of infection ^95^. Another fish rhabdovirus, the SVCV, has been reported to induce accumulation of ROS accompanied by the up-regulation of Nrf2 and its downstream genes (i.e. Heme Oxygenase-1 and thrioredoxin). The overexpression of Nrf2 has been also reported to significantly suppress either entry or replication of several viruses (reviewed in 96), and Shao *et al*. ^96^ also demonstrated that the activation of Nrf2 repressed the replication of SVCV. Therefore, future research could be directed to investigate the implication of the Nrf2 pathway in inhibiting VHSV replication in rainbow trout RBCs.

In summary, this study unveils previously unobserved but important mechanisms for fish nucleated RBCs in the contribution to the defense against a viral aggression not involving RBCs as targets. To our knowledge, this is the first report that implicates fish RBCs as antiviral mediators against viruses targeting other tissues or cells. The recognition of body circulating viruses and the subsequent generation of immune defenses by RBCs may largely contribute to fish survival, given the large volume of RBCs and its rapid and wide distribution to the whole body. We are further investigating if similar mechanisms operate *in vivo*, the molecules that trigger such immune responses or the cellular factors implicated in the interaction with the virus.

## Data availability

The data referenced by this article are under copyright with the following copyright statement: Copyright: © 2018 Nombela I et al.

Data associated with the article are available under the terms of the Creative Commons Zero "No rights reserved" data waiver (CC0 1.0 Public domain dedication). 

F1000Research: Dataset 1. Excel file containing qPCR data. Each sheet contains the raw Ct values for the indicated figure numbers, organized by samples (rows) and genes (columns), 10.5256/f1000research.12985.d192872 ^99^

F1000Research: Dataset 2. Excel file containing the virus titration data. Each sheet contains the virus titer (PFU/mL) results of the indicated figure number, 10.5256/f1000research.12985.d182834 ^100^

F1000Research: Dataset 3. Flow cytometry data. Each folder contains the Flow Cytometry Standard (.fcs) format files. Source data files are organized by figure number, and then by antibody, sample number and condition, 10.5256/f1000research.12985.d192873 ^101^

F1000Research: Dataset 4. Excel file containing the computed peptide spectrum match (PSM) raw data, and the spectra recovered in the iTRAQ 4-plex analysis., 10.5256/f1000research.12985.d182836 ^102^

F1000Research: Dataset 5. Excel file containing the iTRAQ 4-plex quantitative analysis raw data., 10.5256/f1000research.12985.d182837 ^103^

F1000Research: Dataset 6. Excel file containing the densitometry raw data of eIF2α-P and α-Actin western blots. Related uncropped blots are included, 10.5256/f1000research.12985.d192879 ^104^

F1000Research: Dataset 7. Excel file containing DCFDA absorbance raw data, 10.5256/f1000research.12985.d182839 ^105^

## Supplementary material

**Figure S1. Validation of the polyclonal antibody against *Onchorhynchus mykiss* BD1.** Western blotting using the antibody developed in rabbit that recognizes the synthetic BD1 (41aa), in samples from *O. mykiss*: head kidney, muscle, untreated red blood cells (RBCs), VHSV-exposed RBCs, and the synthetic BD1.

**Figure S2. Validation of the polyclonal antibodies against *Onchorhynchus mykiss*** **IFNγ and IFN1.** Left Panel: Prediction of three-dimensional structure of the molecules (by Phyre2). Right panel: Western blotting using the respective antibody. A: Validation of anti-IFNγ antibody. On the left 3D predicted structure of the recombinant protein rIFNγ used for the immunization. On the right the antibody developed in mouse recognizes the rIFNγ. B: Validation of anti-IFN1 antibody, in red: DWIQHHFGHLSAEYLSQ (aa 25-42), the synthetic epitope sequence of IFN1 used for the immunization. On the right, the anti-epitope antibody recognizes the whole molecule expressed in a RT Gill cell line.

**Figure S3. Kinetics of VHSV RNA synthesis in RBCs exposed to the virus.** RBCs were exposed to VHSV with a multiplicity of infection (MOI) of 1 at 14°C. Gene expression was quantified by RT-qPCR at time 0, 1, 3, and 72 hours postexposure (hpe). ( **A**) Random hexamer-primed RT reaction (total RNA) (black bars) and oligo dT primed-RT reaction (mRNA) (dashed bars). Gene expression was normalized against endogenous *ef1α* gene expression and relativized to control cells (non-exposed, time 0, red line) (fold-change). Data is displayed as mean ± SD (n = 3).

**Figure S4. Time course of interferon-inducible antiviral genes *mx* and *pkr* in untreated rainbow trout red blood cells (RBCs).** *mx1-3* (grey) and *pkr* (black) genes expression was quantified at time 0, 3, 6, 24, 72 hours post-ficoll. Gene expression was normalized against eukaryotic 18S rRNA and relativized to control cells (time 0, red line) (fold of increase). Data is displayed as mean ± SD (n = 2). Kruskal-Wallis Test with Dunn´s Multiple Comparison post-hoc test was performed among all conditions.

**Figure S5. Representative flow cytometry dotplots of immune protein responses of VHSV-exposed RBCs.** RBCs were exposed to VHSV at MOI 1, at 14°C, and stained with anti-BD1 ( **A**) and anti-IL8 ( **B**), 72 hpe. Control (non-exposed) and VHSV-exposed RBCs dotplots are shown. Y axis represents side scattering (SSC-A) and X axis FITC fluorescence intensity (FITC-A).

**Figure S6. Pathway network of significantly over-represented GO-terms in VHSV-exposed rainbow trout RBCs protein iTRAQ profiling.** Big nodes represent significantly differentially expressed (down-regulated) proteins that have similar function; edges represent pairwise interactions; small nodes represent the proteins associated to each function. Functional groups are labelled as follows: Blue = proteasome, pink = regulation of RNA stability, light green = cellular catabolic process, dark green= viral process, grey = proteins not associated to any function. A list of all over-represented terms is provided in Table S1.

**Figure S7. NF-kß p65 protein labelling in VHSV-exposed RBCs.** ( **A**) Protein expression levels calculated by the formula MRFI (Mean Relative Fluorescence Intensity) = fluorescence in VHSV-exposed RBCs / fluorescence in non-exposed RBCs, at MOI 1, 10 and 100, 72hpe, at 14°C, relative to control cells (non-exposed, red line). Data represent mean ± SD (n=3). Mann Whitney Test was performed for statistical analysis between the VHSV-exposed cells and control cells. ( **B**) Representative immunofluorescences of control and VHSV-exposed RBCs stained with anti-NF-kß (FITC) and DAPI for nuclei (IF representative of 20 images).

**Table S1. List of significantly over-represented GO-terms in VHSV-exposed rainbow trout RBCs protein iTRAQ profiling.**
