# Supplementary figures and images for: Identification of diverse defense mechanisms in rainbow trout red blood cells in response to halted replication of VHS virus
Source: F1000Res. 2018 Feb 9;6:1958. Originally published 2017 Nov 6. [Version 2] doi: 10.12688/f1000research.12985.2 (PMC5820608; doi:10.12688/f1000research.12985.2)

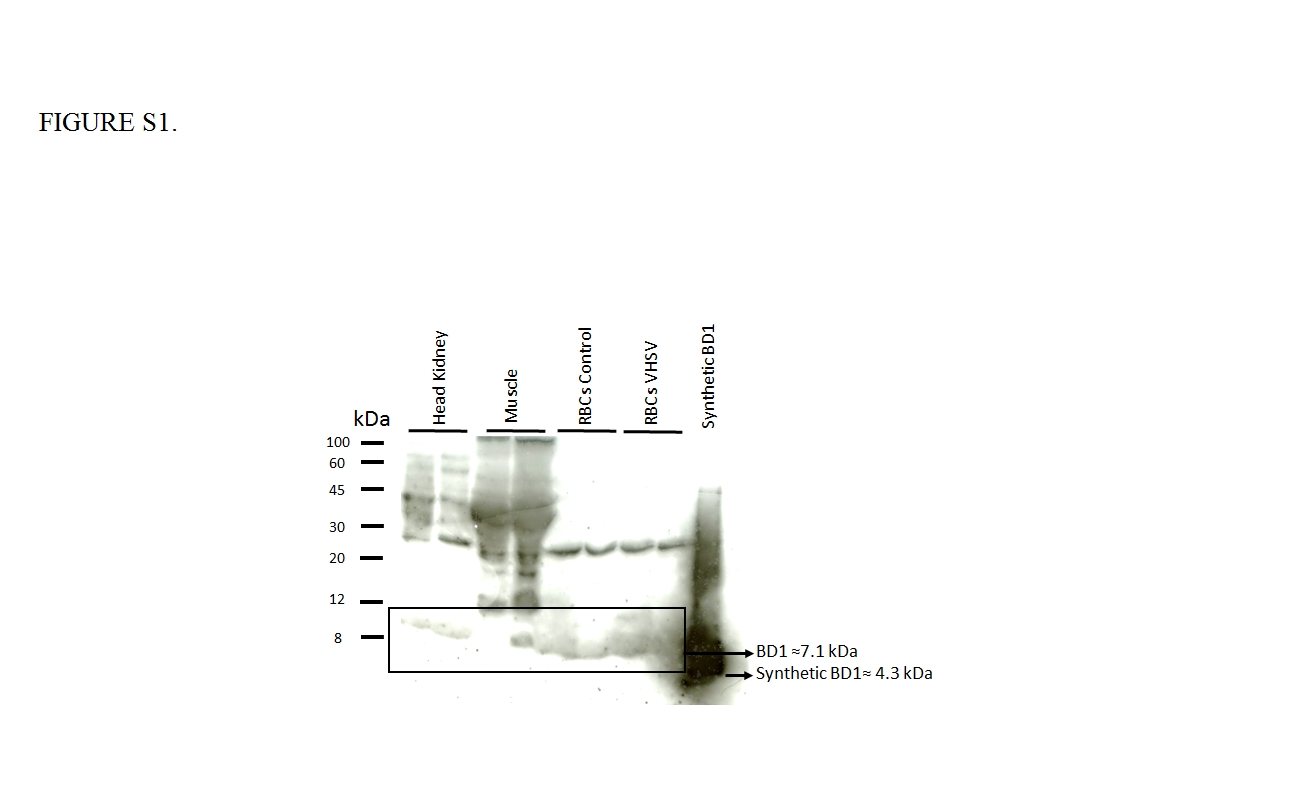

Supplement: Supplementary file 8 [file f1000research-6-15111-s0007.tgz › 3d0fdb29-4241-4c4b-9118-96ef3dd8d8ef.jpg]

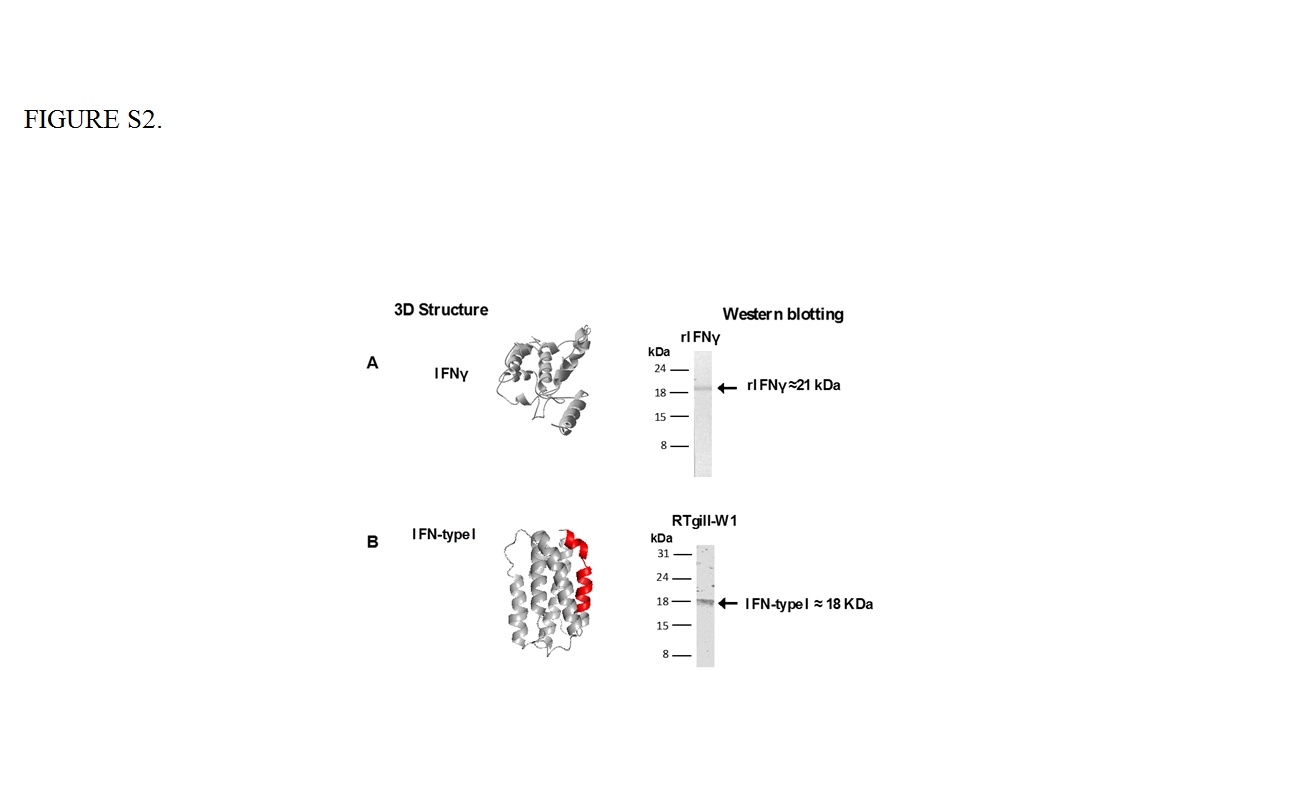

Supplement: Supplementary file 9 [file f1000research-6-15111-s0008.tgz › 96bf4fa6-d425-413d-ba40-dc95aed0eca7.jpg]

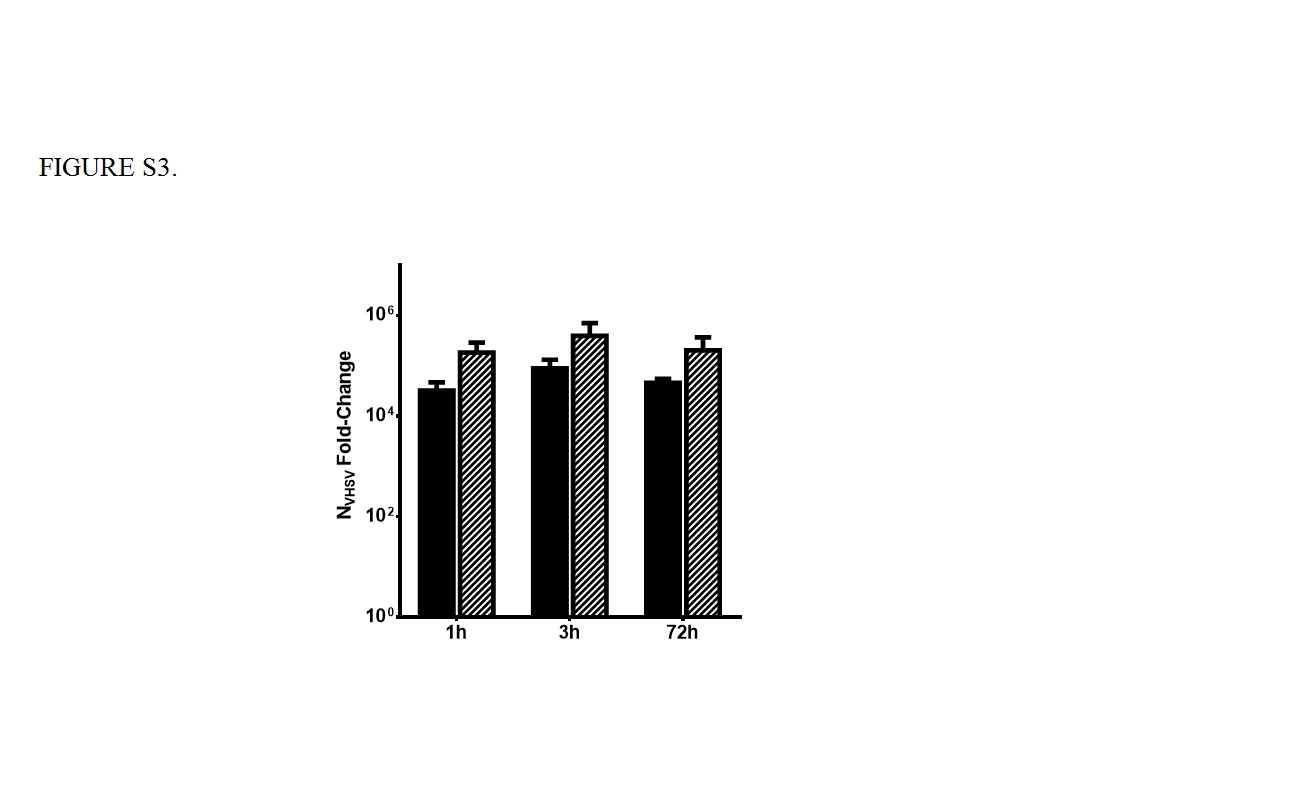

Supplement: Supplementary file 10 [file f1000research-6-15111-s0009.tgz › d7a6bfe5-d359-4050-ac7f-786b2fad681d.jpg]

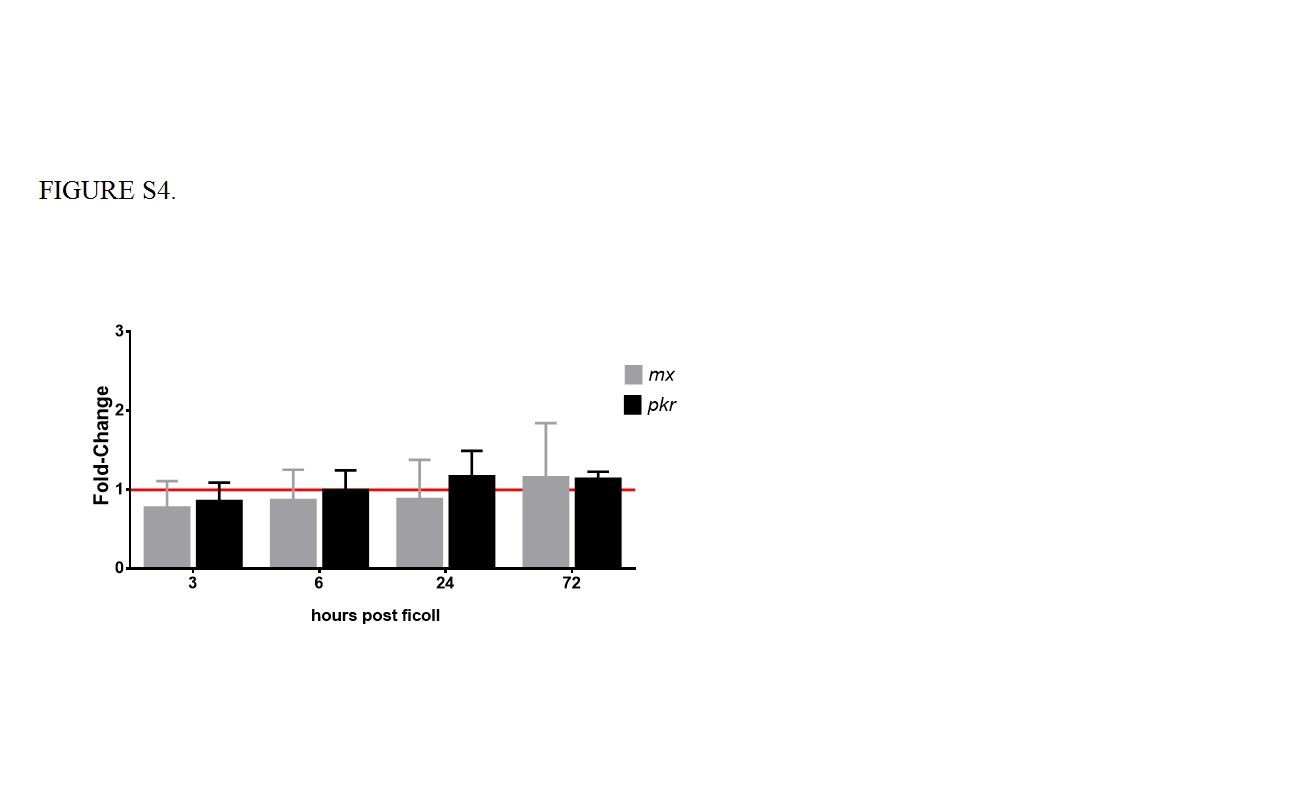

Supplement: Supplementary file 11 [file f1000research-6-15111-s0010.tgz › 982ced46-50cc-44ce-b8f1-0892e770d5bc.jpg]

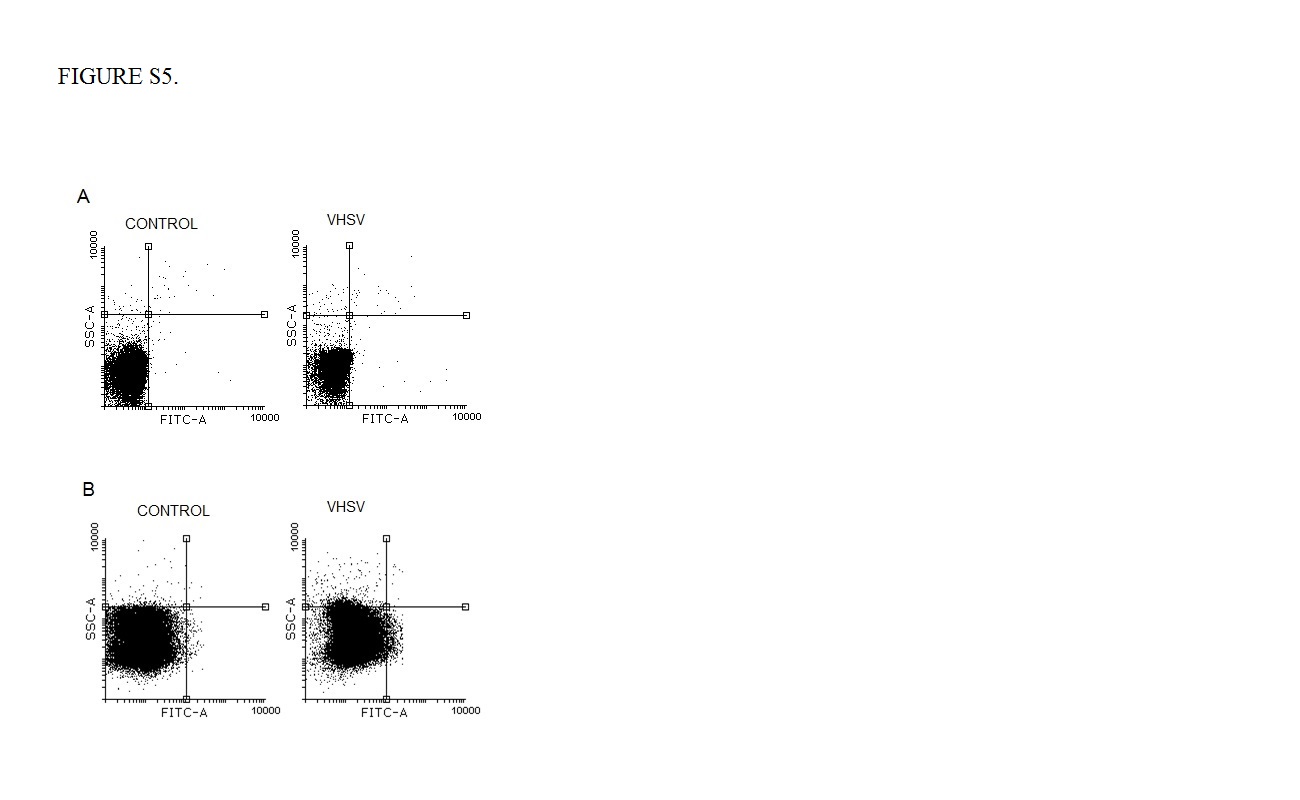

Supplement: Supplementary file 12 [file f1000research-6-15111-s0011.tgz › 8db57f7f-9c45-40b1-af2f-c1741de5cff1.jpg]

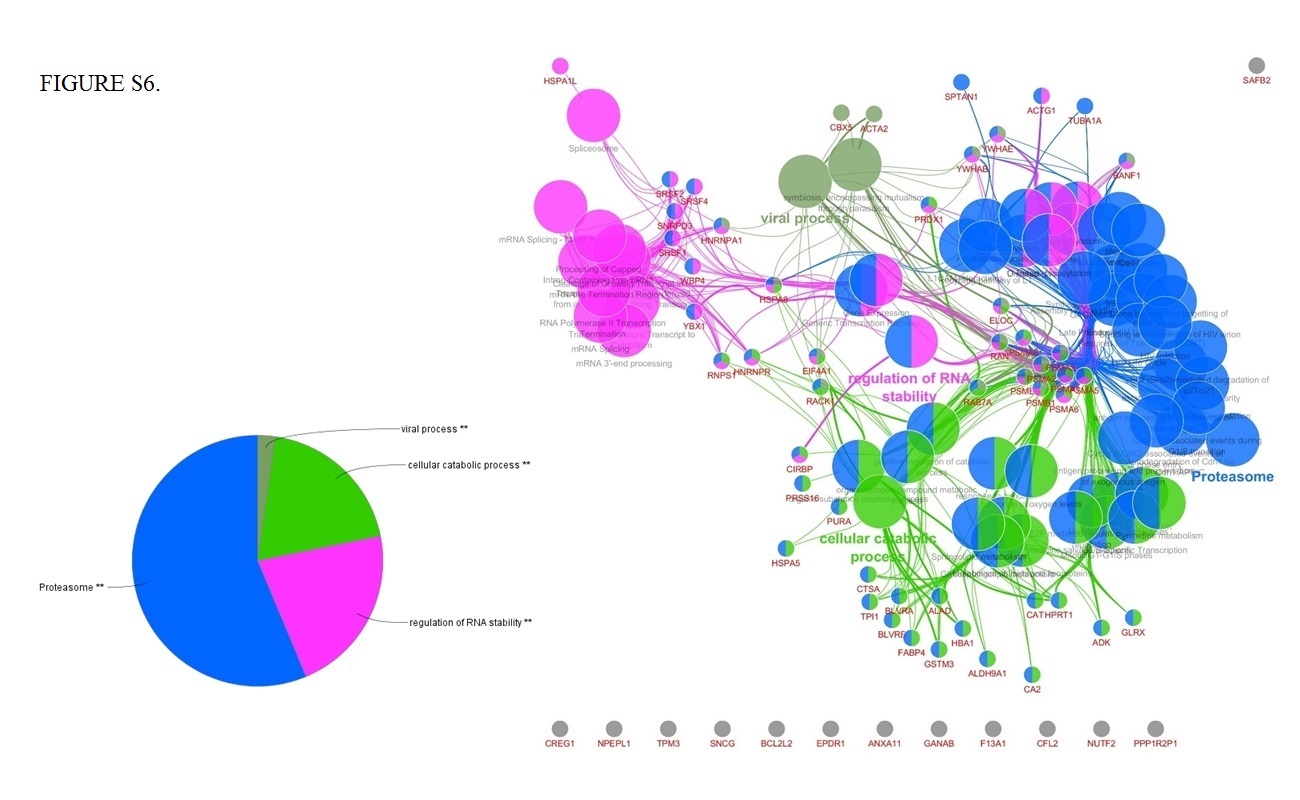

Supplement: Supplementary file 13 [file f1000research-6-15111-s0012.tgz › a95da890-f65a-4b9d-bb37-efa9b1c6f500.jpg]

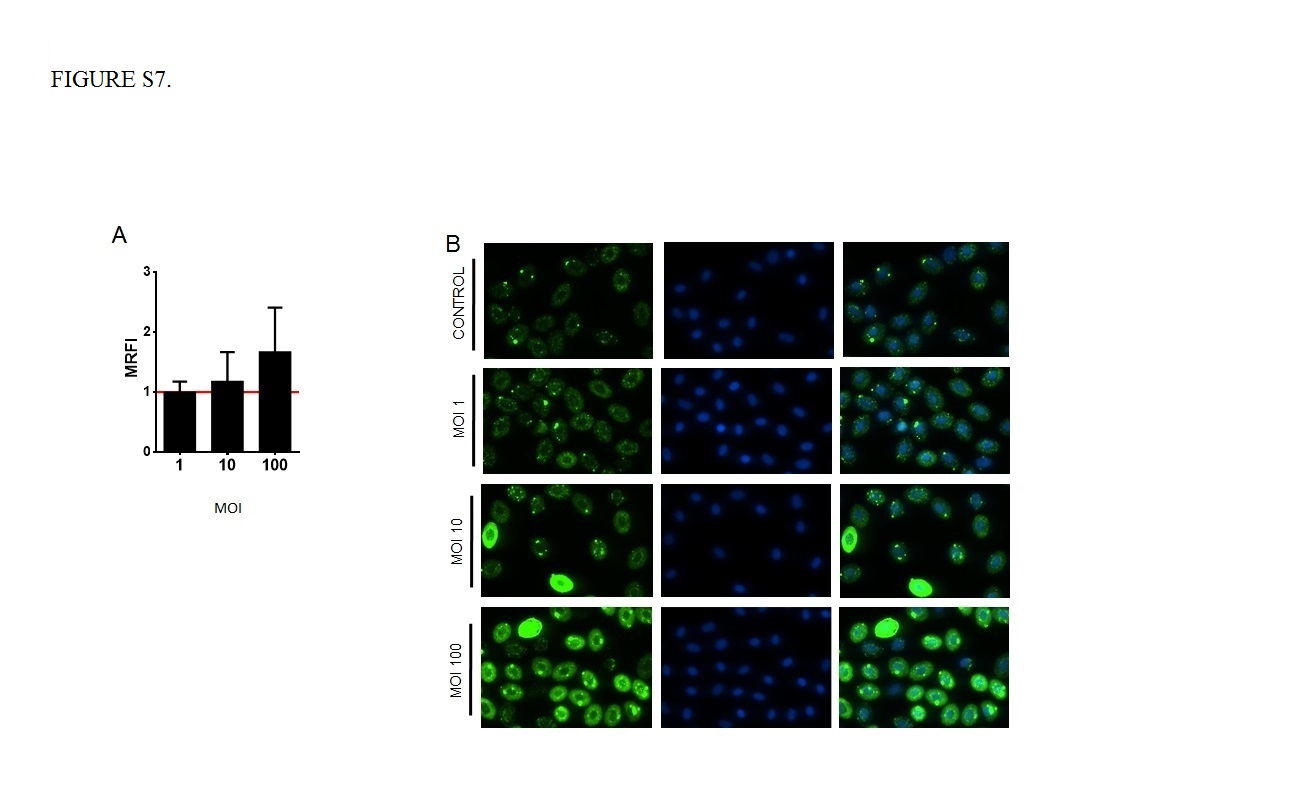

Supplement: Supplementary file 14 [file f1000research-6-15111-s0013.tgz › c37cda34-b26f-4f84-81da-74502ca14c9b.jpg]
