# Supplementary material for: Identification of diverse defense mechanisms in rainbow trout red blood cells in response to halted replication of VHS virus
Source: F1000Res. 2018 Feb 9;6:1958. Originally published 2017 Nov 6. [Version 2] doi: 10.12688/f1000research.12985.2 (PMC5820608; doi:10.12688/f1000research.12985.2)
Supplement: Peer review history

# Peer review: Referee response for version 2

**DOI:** 10.5256/f1000research.15111.r30735

**Published:** 2018-02-19

**Authors:** Johannes M. Dijkstra

**Affiliations:** 1 Institute for Comprehensive Medical Science, Fujita Health University, Toyoake, Japan

The authors performed some additional experiments and rewrote some parts of the manuscript. This improved the article. I am still not overly enthusiastic about the study, because the provided evidence for the several storylines is quite thin. However, I realize that for the type of presented data it is hard to get more convincing results, and believe that also this type of data deserves to be indexed. Therefore, I recommend indexing of the article, although I simultaneously advise the readers to read all reviewer's reports so that they have an understanding of the critical issues of the study.

I have read this submission. I believe that I have an appropriate level of expertise to confirm that it is of an acceptable scientific standard.

# Peer review: Referee response for version 2

**DOI:** 10.5256/f1000research.15111.r30734

**Published:** 2018-02-16

**Authors:** Aleksei Krasnov

**Affiliations:** 1 Nofima AS, Ås, Norway

I am satisfied with the revision.

I have read this submission. I believe that I have an appropriate level of expertise to confirm that it is of an acceptable scientific standard.

# Peer review: Referee response for version 1

**DOI:** 10.5256/f1000research.14081.r27746

**Published:** 2017-12-12

**Authors:** Uwe Fischer

**Affiliations:** 1 Friedrich Loeffler Institute, Greifswald-Insel Riems, Germany

**Summarized statement**

 This is a comprehensive piece of work with a number of interesting experiments.

 However, I suggest to not accepting the paper in its present form.

 To my opinion the authors have not convincingly shown an initial viral replication in RBCs, although this represents their primary experimental basis. All consecutive data are based on this initial assumption and this makes the whole story challengeable.

 In contrast to the assumption of viral replication detection of viral RNA by RT-PCR could have been simply based on inoculated virus that could have been absorbed to the surface of RBCs or internalized by them. In order to prove viral replication de novo synthetized viral RNA or protein expression must be determined.

 In this system viral RNA replication can be determined on the mRNA level. For the RT step e.g. oligo(dT) primers could be used that only bind to the polyadenylated tail of mRNAs. The resulting cDNA must then be thoroughly treated with RNAse. This experiment is essential to make the experiments reliable.

 Alternatively, I suggest modifying the whole story and stressing the obvious responses of RBCs to the virus (avoiding the claim of virus replication). Thus the title could be changed into something like e.g. “Response of rainbow trout RBCs to VHSV”.

**Specific comments**

 Introduction:

 The authors claim: "To compensate for those immune deficiencies, fish have unique phagocytic B lymphocytes".

 This is not fully true since also B lymphocytes of some mammalians can execute phagocytosis.

 They further claim: "To compensate for those immune deficiencies, … and stronger innate immune responses, as shown in survivors of viral infection".

 This is highly speculative and can’t be concluded from a single paper.

 Material and methods:

 The anti-β-defensin (BD1) anti -IFN1 and anti -IFNγ antibodies have not been described or characterized elsewhere. Their suitability can be questioned.

 The anti-Mx3 antiserum has been produced against a predicted Mx peptide. However, I did not find information on the characterization of this antiserum.

 The anti-IL1β and the NKEF antibodies have only been tested against the immunizing peptide and there is no further information if the antibodies react with the respective native proteins.

 The rabbit polyclonal antibody against human NF-κβ p65 has not been shown to be suitable in any fish species. Abcam only declares that it reacts with mouse, rat, chicken, human and Indian muntjac (Heterocephalus glaber). Those species are phylogenetically not closely related to teleosts.

 The authors describe: "Separately, NVHSV RT-qPCR was also used to quantify the viral RNA inside VHSV-exposed RBCs …."

 This is not a proof that virus was inside erythrocytes. The virus could have been simply attached/absorbed to RBCs.

 To show viral replication RT-PCR with mRNA is required.

 "RBCs were fixed with 4% paraformaldehyde (PFA)…"

 Unfixed RBCs should have been used in addition to fixed RBCs in order to distinguish between intracellular and membrane bound virus.

 Results:

 The authors conclude: “…to monitor the replication of VHSV in trout RBCs. … Clearly, the expressions of NVHSV gene were significantly upregulated at 3 hours postexposure.”.

 Again, I can’t support this statement since replication can’t be shown by RT-PCR for an RNA virus. Here simply increasing attachment to RBCs might have occurred which explains lower Cq values (higher amounts of viral RNA, respectively). This needs to be verified on the mRNA level.

 The authors write: “On the other hand, after VHSV enters the cell, the first gene that starts to transcribe is the NVHSV gene,…”.

 This observation has been made in cell cultures that are permissive for VHSV. However, it has not convincingly been shown in this paper that N gene transcription took place in RBCs (see my comments above).

 “NVHSV RT-qPCR also confirmed the presence of viral RNA in VHSV-infected RBCs (Figure 2B).”

 Similarly to the above mentioned, this just says that VHSV was associated with RBCs, but not if the virus was inside RBCs. Attached virus could have resulted in positive RT-PCR results as well.

 To further illustrate my concerns regarding viral replication in RBCs I have a few comments regarding Figure 1:

 The authors write that “The initial VHSV inoculum titer declined ~3-logs after 3 days".

 In Figure 1H, however, I see a 4 log (from 10-to-the-6 to 10-to-the-2) reduction after 3 days (72 hours). 

 As for Figure 1H a negative control is missing where the same inoculum should have been added to the corresponding amount of cell-free medium or, even better, to inactivated (irradiated) erythrocytes. At 14°C, viral titers would probably also drop in the absence of cells (RBCs).

 In Figure 1H the viral titers with RBCs drop within 72 hours by log4. However, in Figure 1D I can see an increase (although minor) in viral titers between 72 hours and 6 days in untreated cells. Is this statistically approved?

 In Figure 1D the authors claim that “VHSV internalization in trout RBCs is NH4Cl-sensitive.” I can’t follow this conclusion. If internalization was NH4Cl-sensitive why it did not fully block virus internalization? The difference between untreated and treated RBCs is about only 2 fold. Is this statistically approved?

 Why the decrease in internalization wasn’t checked 3 hours after exposure when the N gene expression was claimed to be highest in RBCs, and why was it recorded 3 days post exposure when viral titers have been dropped by 4 logs anyway?

 The NH4Cl blocking experiment should have been done with RTG-2 cells as a positive control?

**Ethical statement**

 The anti-IL1β antibody and the NKEF antibodies have been produced by ascitic tumor induction. This method is not in line with European standards of animal welfare.

 Since there are already two reports on this manuscript by two other referees, which I agree on, I will not repeat their objections and suggestions.

I have read this submission. I believe that I have an appropriate level of expertise to confirm that it is of an acceptable scientific standard, however I have significant reservations, as outlined above.

# Response

**Published:** 2018-01-30

**Authors:** Maria del Mar Ortega-Villaizan Romo

**Affiliations:** Universidad Miguel Hernandez, Spain

Dear Dr. Uwe Fischer,

Thank you for your revision and comments on the manuscript. We have included your corrections in the new version of the manuscript hoping that now the manuscript will be suitable for publication.

Please find below the response to your comments:

**_____**

This is a comprehensive piece of work with a number of interesting experiments.

However, I suggest to not accepting the paper in its present form.

To my opinion the authors have not convincingly shown an initial viral replication in RBCs, although this represents their primary experimental basis. All consecutive data are based on this initial assumption and this makes the whole story challengeable.

In contrast to the assumption of viral replication detection of viral RNA by RT-PCR could have been simply based on inoculated virus that could have been absorbed to the surface of RBCs or internalized by them. In order to prove viral replication de novo synthetized viral RNA or protein expression must be determined.

In this system viral RNA replication can be determined on the mRNA level. For the RT step e.g. oligo(dT) primers could be used that only bind to the polyadenylated tail of mRNAs. The resulting cDNA must then be thoroughly treated with RNAse. This experiment is essential to make the experiments reliable.

Response: De novo synthetized viral RNA has been determined by Oligo(dT) RT-qPCR. We have included this result as supplementary Figure (Figure S1). As it can be observed, the kinetics of VHSV mRNA expression closely matched total virus RNA synthesis.

Alternatively, I suggest modifying the whole story and stressing the obvious responses of RBCs to the virus (avoiding the claim of virus replication). Thus the title could be changed into something like e.g. “Response of rainbow trout RBCs to VHSV”.

Response: We appreciate the suggestion, however, in view of other reviewers’ comments, we would like to maintain the title as it is.

**Specific comments**

Introduction:

The authors claim: "To compensate for those immune deficiencies, fish have unique phagocytic B lymphocytes".

This is not fully true since also B lymphocytes of some mammalians can execute phagocytosis.

Response: We have removed this sentence.

They further claim: "To compensate for those immune deficiencies, … and stronger innate immune responses, as shown in survivors of viral infection".

This is highly speculative and can’t be concluded from a single paper.

Response: We have removed this sentence.

Material and methods:

The anti-β-defensin (BD1) anti -IFN1 and anti -IFNγ antibodies have not been described or characterized elsewhere. Their suitability can be questioned.

Response: The antiserum against β-defensin has been validated by our laboratory. Figure S2 depicts western blotting validation for anti-BD1 antibody.  anti -IFN1 and anti -IFNγ antibodies have been validated by Dr. Luis Mercado laboratory. Figure S3 depicts western blotting validation for anti -IFN1 and anti –IFNγ.

The anti-Mx3 antiserum has been produced against a predicted Mx peptide. However, I did not find information on the characterization of this antiserum.

Response: anti-Mx3 antiserum production was described in Chico et al. 2010, Journal of Virology. In Martinez-Lopez et al. 2014 and Ortega-Villaizan et al. 2011 they showed the Mx protein expression in stimulated RTG-2 cells, using anti-Mx3 antibody.

The anti-IL1β and the NKEF antibodies have only been tested against the immunizing peptide and there is no further information if the antibodies react with the respective native proteins.

Response:  For anti-IL1β, in Schmitt et al. 2015 and Rojas et al. 2015, we can find the

expression of IL1β in RTS11 and RTgutGC cells, respectively, by immunofluorescence and western blot. For anti-NKEF, Bethke et al. 2012, show western blotting of NKEF protein in serum of Atlantic salmon. Also Tafalla et al. 2011 show the expression of NKEF in RTS11 as evaluated by western blotting and by flow cytometry.

The rabbit polyclonal antibody against human NF-κβ p65 has not been shown to be suitable in any fish species. Abcam only declares that it reacts with mouse, rat, chicken, human and Indian muntjac (Heterocephalus glaber). Those species are phylogenetically not closely related to teleosts.

Response: We indicated references where this antibody has been used for teleost species:

Encinas P, Garcia-Valtanen P, Chinchilla B, et al.: Identification of multipath genes differentially expressed in pathway-targeted microarrays in zebrafish infected and surviving spring viremia carp virus (SVCV) suggest preventive drug candidates. PLoS One. 2013;8(9):e73553. 24069208 10.1371/journal.pone.0073553 3772095

García-Valtanen P, Martinez-Lopez A, Ortega-Villaizan M, et al.: In addition to its antiviral and immunomodulatory properties, the zebrafish β-defensin 2 (zfBD2) is a potent viral DNA vaccine molecular adjuvant. Antiviral Res. 2014;101:136–47. 24286781 10.1016/j.antiviral.2013.11.009

Abós B, Castro R, González Granja A, et al.: Early activation of teleost B cells in response to rhabdovirus infection. J Virol. 2015;89(3):1768–80. 25410870 10.1128/JVI.03080-14 4300759

The authors describe: "Separately, NVHSV RT-qPCR was also used to quantify the viral RNA inside VHSV-exposed RBCs …."

This is not a proof that virus was inside erythrocytes. The virus could have been simply attached/absorbed to RBCs.

To show viral replication RT-PCR with mRNA is required.

Response: As previously indicated, we have included that result as supplementary Figure (Figure S1). As it can be observed, the kinetics of VHSV mRNA expression closely matched total virus RNA synthesis.

"RBCs were fixed with 4% paraformaldehyde (PFA)…"

Unfixed RBCs should have been used in addition to fixed RBCs in order to distinguish between intracellular and membrane bound virus.

Response: Yes, it is right. However, we have tried to do immune staining of unfixed RBCs and it is very difficult since RBCs are lysed during the antibody incubation.

Results:

The authors conclude: “…to monitor the replication of VHSV in trout RBCs. … Clearly, the expressions of NVHSV gene were significantly upregulated at 3 hours postexposure.”.

Again, I can’t support this statement since replication can’t be shown by RT-PCR for an RNA virus. Here simply increasing attachment to RBCs might have occurred which explains lower Cq values (higher amounts of viral RNA, respectively). This needs to be verified on the mRNA level.

Response: As previously indicated, we have included that result as supplementary Figure (Figure S1). As it can be observed, the kinetics of VHSV mRNA expression closely matched total virus RNA synthesis.

The authors write: “On the other hand, after VHSV enters the cell, the first gene that starts to transcribe is the NVHSV gene,…”.

This observation has been made in cell cultures that are permissive for VHSV. However, it has not convincingly been shown in this paper that N gene transcription took place in RBCs (see my comments above).

“NVHSV RT-qPCR also confirmed the presence of viral RNA in VHSV-infected RBCs (Figure 2B).”

Similarly to the above mentioned, this just says that VHSV was associated with RBCs, but not if the virus was inside RBCs. Attached virus could have resulted in positive RT-PCR results as well.

Response:  The reviewer is correct, RT-qPCR data do not tell if the virus is inside or attached to the cell. But we would like to highlight that Figure 1E shows that in RBCs exposed to UV-inactivated VHSV, Nvhsv levels did not increase in comparison with live VHSV exposed RBCs. This experiment demonstrates that live VHSV replicated early post-exposure, in comparison with UV-inactivated VHSV. In order to emphasize this result we have re-written its explanation.

To further illustrate my concerns regarding viral replication in RBCs I have a few comments regarding Figure 1:

The authors write that “The initial VHSV inoculum titer declined ~3-logs after 3 days".

In Figure 1H, however, I see a 4 log (from 10-to-the-6 to 10-to-the-2) reduction after 3 days (72 hours). 

Response: It was a mistyping error, we have corrected it.

As for Figure 1H a negative control is missing where the same inoculum should have been added to the corresponding amount of cell-free medium or, even better, to inactivated (irradiated) erythrocytes. At 14°C, viral titers would probably also drop in the absence of cells (RBCs).

Response: VHSV titer  in cell-free medium 3 d at 14ºC does not drop more than one log.

In Figure 1H the viral titers with RBCs drop within 72 hours by log4. However, in Figure 1D I can see an increase (although minor) in viral titers between 72 hours and 6 days in untreated cells. Is this statistically approved?

Response: Yes, we observed a minor increase at 6 days post exposure (from  ~ 200 to  ~ 300 PFU/ml), and not statistically significant.

In Figure 1D the authors claim that “VHSV internalization in trout RBCs is NH4Cl-sensitive.” I can’t follow this conclusion. If internalization was NH4Cl-sensitive why it did not fully block virus internalization? The difference between untreated and treated RBCs is about only 2 fold. Is this statistically approved?

Why the decrease in internalization wasn’t checked 3 hours after exposure when the N gene expression was claimed to be highest in RBCs, and why was it recorded 3 days post exposure when viral titers have been dropped by 4 logs anyway?

Response: You are right, the sentence is quite confusing, we have re-written it. We would like to clarify that NH4Cl blocks fusion of viruses to cell membrane, not internalization. On the other hand, the concentration used for NH4Cl (7 mM) was selected since it is non-cytotoxic for RBCs and other cells, such as EPC and RTG-2, but effective for reducing VHSV infectivity by 40% in EPC cells (Mas et al. 2002, Journal of General Virology), similar to the reduction that we observe in RBCs.

The NH4Cl blocking experiment should have been done with RTG-2 cells as a positive control?

Response: We have previously carried out this assay in RTG-2 cells and observed that, at 10 mM NH4Cl, VHSV infection is reduced by 90% in RTG-2 cells, quantified by Nvhsv RT-qPCR. However, a reduction of 50% was observed by Mas et al. 2002 at the same NH4Cl concentration, in EPC cells, although in this case it was quantified by FFU/ml.

**Ethical statement**

The anti-IL1β antibody and the NKEF antibodies have been produced by ascitic tumor induction. This method is not in line with European standards of animal welfare.

Response: Only anti-NKEF, produced at the laboratory of Dr. Luis Mercado (Pontificia Universidad Católica de Valparaíso, Valparaíso, Chile), was developed in ascities fluids, because this antibody was obtained in 2011, when this technique was allowed in Chile. Nowadays, anti-NKEF is no longer produced by ascitic tumor induction, following the Chilean standards for animal welfare.

# Peer review: Referee response for version 1

**DOI:** 10.5256/f1000research.14081.r27745

**Published:** 2017-11-30

**Authors:** Johannes M. Dijkstra

**Affiliations:** 1 Institute for Comprehensive Medical Science, Fujita Health University, Toyoake, Japan

Nombela et al. describe in what they claim to be defense mechanisms in trout red blood cells in response to halted replication of VHSV virus.

**Major comments:**

 The study suffers from the effort to simultaneously answer a few unknowns. Can VHSV infect trout erythrocytes? Does infection by, or exposure to, VHSV virus modify the expression of genes with an anti-virus function? Do those altered gene expression levels have a measurable immune effect (which in this article is measured by the effect on cell line TSS)?

 In my opinion, the authors show understanding of the complexity of those questions, but do not take sufficiently control of them. I am not satisfied with any of the story lines. My main concerns are that it is unclear for many of the presented data (i) whether the enhanced expression of immune molecules are due to RBC infection or due to other stimulations of the RBCs by the virus preparation, (ii) whether the changes in immune molecule expression are due to stimulation by virus preparation or due to time of culturing, and (iii) whether the expression levels of immune molecules reach meaningful levels or are just variation within what could be considered as “non-functional background levels”.

 At the very positive side, the authors addressed an important question, and delivered an honest and elaborate piece of work. Therefore, I will not reject the paper, but I do request the addition of experimental data that in my opinion are necessary for better interpretation of the currently presented data in relation to actual VHS disease.

 The authors should infect rainbow trout cells which they deem (sufficiently representative of) the natural host cells of VHSV, and use the UV-inactivated supernatant for stimulation of trout RBCs and compare the effect on RBC immune molecule expression with the effect after RBC incubation with VHSV. Alternatively, they can use inactivated serum of VHSV-infected trout. My guess is that the released cytokines have a much stronger effect on those erythrocytes than the viruses to which the erythrocytes are hardly receptive.

 The authors should also use those supernatants of natural host cells, or sera from infected trout, for stimulation of TSS cells, and compare the effects quantitatively with those after stimulation with the supernatant of VHSV-exposed erythrocytes.

 The above requested set of experiments (or modifications thereof, depending on the preferences of the authors) should help to quantitatively estimate the direct effect of VHSV on erythrocytes, and the effect of VHSV-stimulated erythrocytes on other cells, in comparison with other routes of immune stimulation during VHSV infection.

****

**Detailed comments:**

 Why did the authors use an MOI of 1? Even if such MOI is achieved, only half of the cells are expected to be infected. In this case the actual MOI for red blood cells probably was far below 1, because the MOI was calculated based on infection of the receptive EPC cells.

 How were the viruses prepared? It seems that they were generated on EPC cells, but the details are important. Namely, other than the viruses, the infected cells also release cytokines which may have a cross-species effect.

 The only presented data that I find convincing for that red blood cells were infected were the experiments shown in Fig. 1E and Fig. 1F, namely after pretreatment with neuraminidase (Fig. 1E) and infection with an MOI=100 (Fig. 1G; even in that case only 1/6 cells shows infection). However, none of the experiments on immune molecule expression was done under those conditions.

 In the experiments, expression levels are compared with those of “control cells”. In some cases those control cells are not specified, while in other cases they are said to be the T=0 cells. However, this does not take into account that also the time of culture can have a significant effect on gene and protein expression levels. Most of the expression level effects reported in this article are quite small (e.g. from very low to only two-fold higher), and a possible “culture-time effect” should have been excluded.

 The introduction should give detailed descriptions of what is known or unknown (i) about natural target cells and receptors used by VHSV for infection, and (ii) about fish erythrocytes and to what extent they have a normal metabolism. The introduction should also give an indication of the abundance of erythrocytes, which is relevant because many small amounts of cytokine could make a big amount, and also because any “intelligent” virus will do its best to avoid interaction with this abundant and for the virus non-productive cell type.

 In the title, shouldn’t it be “in response to halted replication of VHS virus.”?

 In the abstract and in the text: “after 6 hours postexposure” is double.

 In the abstract, in the sentence “Co-culture assays of RBCs with TSS”, it should be made clear that those RBCs were stimulated with UV-inactivated VHSV.

 In the introduction, a number of speculations are presented as facts:

- Fish poikilothermic nature results in a delayed antigen affinity maturation, memory and lymphocyte proliferation.
- Fish have unique phagocytic B lymphocytes. (later than the reference, also mammalian B cells with phagocytic ability have been found)
- Fish have stronger innate immune responses.
- To compensate for those immune deficiencies, fish have unique phagocytic B lymphocytes and stronger innate immune responses.

 

 I don’t understand the “Thus” in the sentence “Thus, fish RBCs generate a wide variety of immune-related gene transcripts when viruses highly replicate inside them”.

 In Fig. 1A, how was the PCR value for N gene determined at T=0? Was that before or after addition of the viruses, and could the difference between T=0 and the other time points be explained by amplification from RNA in virions?

 Fig. 1B seems to argue against the assumption that the RNAs amplified in Fig. 1A were derived from an infection (see also my previous point). In addition, although the relative comparison between the RTG2 and RBC results as presented in Fig. 1B should be OK, it is unclear to me from the materials and methods section how the absolute quantitative statement “However, a ratio of 2 was observed in RBCs, compared to the ratio of 8 found in RTG-2 cells, at 1 and 3 hpe (Figure 1B)” can be made.

 I am not convinced that Fig. 1D is evidence for replication between days 3 and 6, since the titer goes down >5000-fold from day 0 to day 3, and then stays very low. The authors should make clearer whether they feel that the small increase in virus titer between days 3 and 6 is only suggestive of virus replication, or that such replication is supported by proper statistics.

 As for the NH4Cl effect observed in Fig. 1D. Can a chemical effect of NH4CL on the integrity of virions stuck to the outside of RBCs be excluded from explaining the results? Furthermore, I would like the authors to elaborate, possibly in the introduction section, on endocytosis in regard to erythrocytes and VHSV infection. Could it be that only immature erythrocytes are expected to display efficient endocytosis, and might the Fig. 1F result be explained by differences in erythrocyte subpopulations?

 I don’t understand the sentence “As a result, the VHSV RNA inside RBCs was increased about ten times at 3 hpe”, because the increase seems to be from around 0.6 to 3.4, which is closer to a six-fold increase.

 For discussion of the Fig. 1F result, the authors should explain the intracellular organization of RBCs (which are unusual cells), and where VHSV is expected. The sentence “along the cytoplasm and nucleus” can’t be understood, and gives the impression that the authors do not know where to expect (normal) cytoplasm in RBCs. Although they observe “along the nucleus”, which I think is the correct observation, the authors discuss the possibility of N protein being present in the nucleus. It is not wrong to present that as a possibility to partially explain their observations, but the authors should declare clearer that their observations do not necessitate that N protein is present in the nucleus. To superficial readers it now looks as if they claim detection of N protein in the RBC nucleus.

 In Fig. 3 legend, the B and C order should be altered.

 (writing error) were co-culture with > were co-cultured with

 In the Transwell system, the authors tried to get rid of RBC-attached virions with a single non-stringent wash. I doubt that such was sufficient for complete removal, and in the following 24 hours of co-incubation some virions or viral products may have diffused to the TSS cells.

 As for Fig. 4. Is it OK to assume linear regression based on only two time points? Wouldn’t it be more proper to indicate all individual observations with dots? What is a 0-fold increase? In Figs. 4A and 4C, all three lines need explanation.

 I can’t trust the claims based on Fig. 8. In Fig. 8A, why were protein amounts loaded in the Control and VHSV lanes so different? I don’t believe that densiometry analysis technique for Western blot bands is sufficiently sensitive, especially not if comparing different ranges of band densities, to reliably claim an about 15% difference as done in Fig. 8B. In addition, for densiometry analysis, the Fig. 8A Actin blot is a horrible result because only half of the lane was properly exposed to the Western treatment. If the Fig. 8A result truly is a “representative” result, as claimed by the authors, the Fig. 8 based conclusions can’t be taken seriously.

 Trout erythrocytes are known to express MHC class I (Dijkstra *et al.* (2003) ^1^; Sarder *et al*. (2003) ^2^). Because MHC class I is a molecule upregulated during virus infection, it would be interesting to see the effect on its expression in RBCs by exposure to VHSV. Likewise, and especially because Nombela et al. discuss the proteasome, it would be interesting to see the regulation of the genes for the immuno-proteasome specific subunits.

I have read this submission. I believe that I have an appropriate level of expertise to confirm that it is of an acceptable scientific standard, however I have significant reservations, as outlined above.

## References

[1] DijkstraJKöllnerBAoyagiKSawamotoYKurodaAOtotakeMNakanishiTFischerU: The rainbow trout classical MHC class I molecule Onmy-UBA*501 is expressed in similar cell types as mammalian classical MHC class I molecules. *Fish & Shellfish Immunology*.2003;14(1) : 10.1006/fsim.2001.0407 1-23 10.1006/fsim.2001.0407 12547623

[2] SarderMRFischerUDijkstraJMKiryuIYoshiuraYAzumaTKöllnerBOtotakeM: The MHC class I linkage group is a major determinant in the in vivo rejection of allogeneic erythrocytes in rainbow trout (Oncorhynchus mykiss). *Immunogenetics*.2003;55(5) : 10.1007/s00251-003-0587-4 315-24 10.1007/s00251-003-0587-4 12879308

# Response

**Published:** 2018-01-30

**Authors:** Maria del Mar Ortega-Villaizan Romo

**Affiliations:** Universidad Miguel Hernandez, Spain

Dear Dr. Johannes M. Dijkstra,

 We appreciate very much your detailed revision and constructive comments and suggestions on the manuscript. We have included your corrections in the new version of the manuscript hoping that now the manuscript will be suitable for publication.

 Please find below the response to your comments:

 ______

**Major comments:**

 The study suffers from the effort to simultaneously answer a few unknowns. Can VHSV infect trout erythrocytes? Does infection by, or exposure to, VHSV virus modify the expression of genes with an anti-virus function? Do those altered gene expression levels have a measurable immune effect (which in this article is measured by the effect on cell line TSS)?

 In my opinion, the authors show understanding of the complexity of those questions, but do not take sufficiently control of them. I am not satisfied with any of the story lines. My main concerns are that it is unclear for many of the presented data (i) whether the enhanced expression of immune molecules are due to RBC infection or due to other stimulations of the RBCs by the virus preparation, (ii) whether the changes in immune molecule expression are due to stimulation by virus preparation or due to time of culturing, and (iii) whether the expression levels of immune molecules reach meaningful levels or are just variation within what could be considered as “non-functional background levels”.

 At the very positive side, the authors addressed an important question, and delivered an honest and elaborate piece of work. Therefore, I will not reject the paper, but I do request the addition of experimental data that in my opinion are necessary for better interpretation of the currently presented data in relation to actual VHS disease.

 The authors should infect rainbow trout cells which they deem (sufficiently representative of) the natural host cells of VHSV, and use the UV-inactivated supernatant for stimulation of trout RBCs and compare the effect on RBC immune molecule expression with the effect after RBC incubation with VHSV. Alternatively, they can use inactivated serum of VHSV-infected trout. My guess is that the released cytokines have a much stronger effect on those erythrocytes than the viruses to which the erythrocytes are hardly receptive.

 The authors should also use those supernatants of natural host cells, or sera from infected trout, for stimulation of TSS cells, and compare the effects quantitatively with those after stimulation with the supernatant of VHSV-exposed erythrocytes.

 The above requested set of experiments (or modifications thereof, depending on the preferences of the authors) should help to quantitatively estimate the direct effect of VHSV on erythrocytes, and the effect of VHSV-stimulated erythrocytes on other cells, in comparison with other routes of immune stimulation during VHSV infection.

Response: This is an interesting suggestion. To answer this question we have carried out an experiment where RTG-2 cells were treated with UV-inactivated VHSV during 24h, after that UV-VHSV was removed and cells were culture 24h in RPMI fresh medium. This conditioned medium was used to stimulate rainbow trout RBCs, during 24h. RTG-2 is a known cell line susceptible to VHSV infection and with a high interferon response to VHSV, therefore we considered it as a proper cell line for this experiment. Please see the results in Figure 4D.

In relation to the last sentence, we did not proceed with that assay since the results obtained with RTG-2 conditioned media on RBCs were conclusive.

**Detailed comments:**

 Why did the authors use an MOI of 1? Even if such MOI is achieved, only half of the cells are expected to be infected. In this case the actual MOI for red blood cells probably was far below 1, because the MOI was calculated based on infection of the receptive EPC cells.

Response: We have also infected RBCs with lower and higher MOIs, as can be observed in Figure 1C, in order to monitor course of infection. However, for immune response experiments we decided to use MOI 1 since over this value more defecting interfering virus will be present. Moreover, MOI1 is already a high titer in experiments of immune response to the infection.

 How were the viruses prepared? It seems that they were generated on EPC cells, but the details are important. Namely, other than the viruses, the infected cells also release cytokines which may have a cross-species effect.

Response: VHSV was prepared in EPC cells as indicated in Methods. As suggested, we have expanded the details about it. As Dr. Dijkstra comments, we are aware that the clarified supernatant contains cytokines that may have a cross-species effect. However, the volume of supernatant/inoculum needed to infect may be less than 0.01 ul, since we usually have virus stock titter of 10 ^7 ^ffu/ml. Therefore, cytokines from EPC cells are diluted 1:100.   

 The only presented data that I find convincing for that red blood cells were infected were the experiments shown in Fig. 1E and Fig. 1F, namely after pretreatment with neuraminidase (Fig. 1E) and infection with an MOI=100 (Fig. 1G; even in that case only 1/6 cells shows infection). However, none of the experiments on immune molecule expression was done under those conditions.

Response:  Yes, MOI values over 100 are needed to detect VHSV inside RBCs by immunofluorescence. However, MOI1 is already a high titer in experiments of immune response, since one or few viral particles per cell can induce detectable immune response.

 In the experiments, expression levels are compared with those of “control cells”. In some cases those control cells are not specified, while in other cases they are said to be the T=0 cells. However, this does not take into account that also the time of culture can have a significant effect on gene and protein expression levels. Most of the expression level effects reported in this article are quite small (e.g. from very low to only two-fold higher), and a possible “culture-time effect” should have been excluded.

Response: For all the experiments, except for time course assays, control cells are uninfected cells culture in RPMI 2%FBS (viral infection medium), in the same plate as infected cells, and incubated the same time. To better explain it, we have indicated it in each figure legend. In the case of time course experiment (Figure 2), control cells refer to time=0h.  For this assay, in order to evaluate the culture-time effect in the expression of *mx *and *pkr *genes, we have analysed gene expression in non-stimulated RBCs along the time course and observed that it does not change during the three days of culture ex vivo. We have added a Supplementary figure with these results (Figure S4), and commented it in the manuscript.

 The introduction should give detailed descriptions of what is known or unknown (i) about natural target cells and receptors used by VHSV for infection, and (ii) about fish erythrocytes and to what extent they have a normal metabolism. The introduction should also give an indication of the abundance of erythrocytes, which is relevant because many small amounts of cytokine could make a big amount, and also because any “intelligent” virus will do its best to avoid interaction with this abundant and for the virus non-productive cell type.

Response: As advised, we have include information about VHSV targets and cell receptors in the introduction. About RBCs metabolism, we have talked about oxidative stress in the discussion. About the last item (“abundance of erythrocytes, which is relevant because many small amounts of cytokine could make a big amount”), we have talked about it the introduction and discussion.

 In the title, shouldn’t it be “in response to halted replication of VHS virus.”?

Response: Yes, we have corrected it.

 In the abstract and in the text: “after 6 hours postexposure” is double.

Response: We have corrected it.

 In the abstract, in the sentence “Co-culture assays of RBCs with TSS”, it should be made clear that those RBCs were stimulated with UV-inactivated VHSV.

Response: Yes, we have added it.

 In the introduction, a number of speculations are presented as facts:

- Fish poikilothermic nature results in a delayed antigen affinity maturation, memory and lymphocyte proliferation.

Response: We have deleted it to avoid misunderstanding.

- Fish have unique phagocytic B lymphocytes. (later than the reference, also mammalian B cells with phagocytic ability have been found)

Response: We have deleted it to avoid misunderstanding.

- Fish have stronger innate immune responses.

Response: We have deleted it to avoid misunderstanding.

- To compensate for those immune deficiencies, fish have unique phagocytic B lymphocytes and stronger innate immune responses.

Response: We have deleted it to avoid misunderstanding.

 I don’t understand the “Thus” in the sentence “Thus, fish RBCs generate a wide variety of immune-related gene transcripts when viruses highly replicate inside them”.

Response: We have deleted it.

 In Fig. 1A, how was the PCR value for N gene determined at T=0? Was that before or after addition of the viruses, and could the difference between T=0 and the other time points be explained by amplification from RNA in virions?

Response: Control cells are cells non-exposed to the virus, at t=0. We have better explained it in figure legend.

 Fig. 1B seems to argue against the assumption that the RNAs amplified in Fig. 1A were derived from an infection (see also my previous point). In addition, although the relative comparison between the RTG2 and RBC results as presented in Fig. 1B should be OK, it is unclear to me from the materials and methods section how the absolute quantitative statement “However, a ratio of 2 was observed in RBCs, compared to the ratio of 8 found in RTG-2 cells, at 1 and 3 hpe (Figure 1B)” can be made.

Response: It is not clear to me why Fig. 1B argues that the RNAs amplified in Fig. 1A were derived from an infection. Please take into account that Fig. 1A is in log scale and the Nvhsv RNA level increment in RBCs at 3 hpe was low.

About N:G genes ratio, which indicates RNA virus transcription, it was calculated as 2 ^−^ ^ΔΔ^ ^Ct^ N _VHSV_: 2 ^−^ ^ΔΔ^ ^Ct^ G _VHSV_. We have indicated it in the Figure 1B legend.

 I am not convinced that Fig. 1D is evidence for replication between days 3 and 6, since the titer goes down >5000-fold from day 0 to day 3, and then stays very low. The authors should make clearer whether they feel that the small increase in virus titer between days 3 and 6 is only suggestive of virus replication, or that such replication is supported by proper statistics.

Response: This result is not statistically significant. VHSV titers are maintained in RBCs but do not increase. We have explained it in the manuscript.

 As for the NH4Cl effect observed in Fig. 1D. Can a chemical effect of NH4CL on the integrity of virions stuck to the outside of RBCs be excluded from explaining the results? Furthermore, I would like the authors to elaborate, possibly in the introduction section, on endocytosis in regard to erythrocytes and VHSV infection. Could it be that only immature erythrocytes are expected to display efficient endocytosis, and might the Fig. 1F result be explained by differences in erythrocyte subpopulations?

Response: 7mM NH4Cl slightly increases cell pH and therefore inhibits endosome acidification. Thus, virus is kept into the endosome and not released into the cytoplasm. On the other hand, high alkaline pH (about 9) is needed to remove virus binded to cytoplasmic membrane. With 7mM NH4Cl, pH is only slightly increased to 7.5.

In relation to RBCs endocytosis and virus endocytosis, as advised, we have talked about it in the introduction. However, in relation to the differences of endocytosis among RBCs subpopulations, it would be very interesting to evaluate but we do not have any evidence or reference about it.

 I don’t understand the sentence “As a result, the VHSV RNA inside RBCs was increased about ten times at 3 hpe”, because the increase seems to be from around 0.6 to 3.4, which is closer to a six-fold increase.

Response: This comment refers to Figure 1E. Yes, we have now specified that 10 times increment at 3 hpe is in relation to VHSV-UV treated cells.

 For discussion of the Fig. 1F result, the authors should explain the intracellular organization of RBCs (which are unusual cells), and where VHSV is expected. The sentence “along the cytoplasm and nucleus” can’t be understood, and gives the impression that the authors do not know where to expect (normal) cytoplasm in RBCs. Although they observe “along the nucleus”, which I think is the correct observation, the authors discuss the possibility of N protein being present in the nucleus. It is not wrong to present that as a possibility to partially explain their observations, but the authors should declare clearer that their observations do not necessitate that N protein is present in the nucleus. To superficial readers it now looks as if they claim detection of N protein in the RBC nucleus.

Response: Yes, thank you for your observation. After your observation, and in order to avoid misunderstandings, we prefer to eliminate the discussion about the localization of the N protein in the nucleus, and show it as an observation of intracellular localization.

 In Fig. 3 legend, the B and C order should be altered.

Response: We have corrected it in figure 3 legend.

 (writing error) were co-culture with > were co-cultured with

Response: We have corrected it.

 In the Transwell system, the authors tried to get rid of RBC-attached virions with a single non-stringent wash. I doubt that such was sufficient for complete removal, and in the following 24 hours of co-incubation some virions or viral products may have diffused to the TSS cells.

Response: Yes, reviewer is right. However, more stringent washing conditions resulted to be cytotoxic for RBCs.

 As for Fig. 4. Is it OK to assume linear regression based on only two time points? Wouldn’t it be more proper to indicate all individual observations with dots? What is a 0-fold increase? In Figs. 4A and 4C, all three lines need explanation.

Response: We have included individual observations and separated each line. In relation to your question about 0 fold-increase, for that point, TSS vig1 gene expression is 0.15 fold.

 I can’t trust the claims based on Fig. 8. In Fig. 8A, why were protein amounts loaded in the Control and VHSV lanes so different? I don’t believe that densiometry analysis technique for Western blot bands is sufficiently sensitive, especially not if comparing different ranges of band densities, to reliably claim an about 15% difference as done in Fig. 8B. In addition, for densiometry analysis, the Fig. 8A Actin blot is a horrible result because only half of the lane was properly exposed to the Western treatment. If the Fig. 8A result truly is a “representative” result, as claimed by the authors, the Fig. 8 based conclusions can’t be taken seriously.

Response: We have replaced it by a better Western Blot.

 Trout erythrocytes are known to express MHC class I (Dijkstra *et al.* (2003) ^1^; Sarder *et al*. (2003) ^2^). Because MHC class I is a molecule upregulated during virus infection, it would be interesting to see the effect on its expression in RBCs by exposure to VHSV. Likewise, and especially because Nombela et al. discuss the proteasome, it would be interesting to see the regulation of the genes for the immuno-proteasome specific subunits.

Response: Yes, it is an interesting observation. In fact we are working on it and the results wil be published in a different manuscript.

# Peer review: Referee response for version 1

**DOI:** 10.5256/f1000research.14081.r27680

**Published:** 2017-11-27

**Authors:** Aleksei Krasnov

**Affiliations:** 1 Nofima AS, Ås, Norway

The paper reports studies with red blood cells (RBC) of rainbow trout infected with Viral Haemorrhagic Septicaemia virus (VHSV). RBC most likely do not support propagation of VHSV and immune responses to the pathogen were small by magnitude. Still, experiments and analyses were well designed and implemented, applied diverse methods and therefore publication will be useful and interesting for experts in the area.

**Major comment**

 Suppression of innate antiviral immunity in infected RBC is included in the abstract as one of the key findings. However, of several genes analysed with qPCR only ifn1 showed down-regulation and only at one time-point. Other genes exhibited at best a slight tendency and differences from control were small. Reference to high variation is not convincing and does not overcome the lack of significance. Delete sentence “It is noteworthy to highlight the elevated inter-individual variability found in trout RBCs immune response, for most of the proteins and genes assayed, which could prevent to obtain statistical significance in most of the cases although regulations were apparent” (pages 9-10), this statement is trivial. M&M do not tell if each RBC culture was from an individual animal. If not, then variation was technical by character suggesting problems with methods. I also suggest to delete or at least shorten discussion of viral suppression of IFN-dependent responses in fish (page 16, 1 ^st^ paragraph). I would emphasize strong induction of ROS scavengers as most impressive result of this study.

**Specific comments**

- Figure 2. Indicate method in the legend – qPCR? Change label of Y-axis: fold instead of fold of increase.
- Figure 4. The number of replicates (n = 6) is too small for regression and correlation analyses. I strongly suggest to plot empirical data, trend lines alone are not convincing. Judging from the figures, ifn1 levels were in the range from 0 to 15. Units should be explained. Furthermore, it is unclear how such differences was achieved taking into account minor responses of ifn1 to IHNV in trout RBC.
- Figure 10. Explain grey and black bars in the legend.
- Page 16. Delete paragraph “It is noticeable that the iTRAQ-based protein…” – no need to explain that proteomic analyses fail to detect low abundance proteins including cytokines.
- Page 17, 1 ^st^ paragraph. NFkB can be mentioned but extensive discussion is not warranted since study did not produce any experimental data for this gene or protein.
- “It is evident that the antiviral response of RBCs is low compared to other cells of the immune system” – this statement is wrong. Virus infected fish RBC develop immune responses of huge magnitude. “Inter-individual variability” – if RBC cultures represented individuals, this must be explicitly stated in M&M (see comment above). Given small number of replicates, discussion of high and low responders is not supported with data.

I have read this submission. I believe that I have an appropriate level of expertise to confirm that it is of an acceptable scientific standard, however I have significant reservations, as outlined above.

# Response

**Published:** 2018-01-30

**Authors:** Maria del Mar Ortega-Villaizan Romo

**Affiliations:** Universidad Miguel Hernandez, Spain

Dear Dr. Aleksei Krasnov,

 We appreciate very much your thorough revision and positive and constructive comments on the manuscript. We have included your corrections and suggestions in the new version of the manuscript hoping that now the manuscript will be suitable for publication.

 Please find below the response to your comments:

**_____**

 The paper reports studies with red blood cells (RBC) of rainbow trout infected with Viral Haemorrhagic Septicaemia virus (VHSV). RBC most likely do not support propagation of VHSV and immune responses to the pathogen were small by magnitude. Still, experiments and analyses were well designed and implemented, applied diverse methods and therefore publication will be useful and interesting for experts in the area.

**Major comment**

 Suppression of innate antiviral immunity in infected RBC is included in the abstract as one of the key findings. However, of several genes analysed with qPCR only ifn1 showed down-regulation and only at one time-point. Other genes exhibited at best a slight tendency and differences from control were small. Reference to high variation is not convincing and does not overcome the lack of significance. Delete sentence “It is noteworthy to highlight the elevated inter-individual variability found in trout RBCs immune response, for most of the proteins and genes assayed, which could prevent to obtain statistical significance in most of the cases although regulations were apparent” (pages 9-10), this statement is trivial.

Response: We have deleted this sentence in order to avoid misunderstanding.

 M&M do not tell if each RBC culture was from an individual animal. If not, then variation was technical by character suggesting problems with methods.

Response: We have indicated in Methods, Animals section, the following: The number of individuals used is indicated by an “n” in each experiment.

 I also suggest to delete or at least shorten discussion of viral suppression of IFN-dependent responses in fish (page 16, 1 ^st^ paragraph).

Response: As advised, we have shortened the discussion of viral suppression of IFN-dependent responses in fish (page 16, 1st paragraph).

 I would emphasize strong induction of ROS scavengers as most impressive result of this study.

Response: As advised, we have emphasize the discussion related to ROS and antioxidant response in RBCs.

**Specific comments**

- Figure 2. Indicate method in the legend – qPCR? Change label of Y-axis: fold instead of fold of increase.

Response: We have indicated it as advised, in Figure 2 and in other figures as well.

 Figure 4. The number of replicates (n = 6) is too small for regression and correlation analyses. I strongly suggest to plot empirical data, trend lines alone are not convincing. Judging from the figures, ifn1 levels were in the range from 0 to 15. Units should be explained. Furthermore, it is unclear how such differences was achieved taking into account minor responses of ifn1 to IHNV in trout RBC. 

Response: As indicated we have included the individual values. The ifn1 response is normally downregulated in RBCs after VHSV-exposure. However, in a few individuals (outliers) we could find ifn1 upregulation, which correlated with the upregulation in TPS-2.

- Figure 10. Explain grey and black bars in the legend.

Response: We have corrected it and explained it.

- Page 16. Delete paragraph “It is noticeable that the iTRAQ-based protein…” – no need to explain that proteomic analyses fail to detect low abundance proteins including cytokines.

Response: We have deleted this sentence in order to avoid misunderstanding.

 Page 17, 1 ^st^ paragraph. NFkB can be mentioned but extensive discussion is not warranted since study did not produce any experimental data for this gene or protein.

Response: As advised, we have shortened the discussion related to NFkB.  

- “It is evident that the antiviral response of RBCs is low compared to other cells of the immune system” – this statement is wrong. Virus infected fish RBC develop immune responses of huge magnitude. “Inter-individual variability” – if RBC cultures represented individuals, this must be explicitly stated in M&M (see comment above). Given small number of replicates, discussion of high and low responders is not supported with data.

Response: As indicated before, we have indicated in Methods, Animals section, the following: The number of individuals used is indicated by an “n” in each experiment. Separately, in order to avoid misunderstanding we have eliminated the sentence “It is evident that the antiviral response of RBCs is low compared to other cells of the immune system”, “Inter-individual variability” and high and low responders discussion.
